# Surface‐Grafted Biocompatible Polymer Conductors for Stable and Compliant Electrodes for Brain Interfaces

**DOI:** 10.1002/adhm.202402215

**Published:** 2024-07-16

**Authors:** Rachel Blau, Samantha M. Russman, Yi Qie, Wade Shipley, Allison Lim, Alexander X. Chen, Audithya Nyayachavadi, Louis Ah, Abdulhameed Abdal, Guillermo L. Esparza, Samuel J. Edmunds, Ritwik Vatsyayan, Sean P. Dunfield, Moumita Halder, Jesse V. Jokerst, David P. Fenning, Andrea R. Tao, Shadi A. Dayeh, Darren J. Lipomi

**Affiliations:** ^1^ Aiiso Yufeng Li Family Department of Chemical and Nano Engineering University of California, San Diego 9500 Gilman Drive La Jolla CA 92093‐0448 USA; ^2^ Department of Bioengineering University of California, San Diego 9500 Gilman Drive La Jolla CA 92093‐0448 USA; ^3^ Materials Science and Engineering Program University of California, San Diego 9500 Gilman Drive La Jolla CA 92093‐0418 USA; ^4^ Department of Mechanical and Aerospace Engineering University of California, San Diego 9500 Gilman Drive La Jolla CA 92093‐0448 USA; ^5^ Department of Electrical and Computer Engineering University of California, San Diego 9500 Gilman Drive La Jolla CA 92093‐0448 USA

**Keywords:** neural interface, PEDOT, polymer brushes, self‐assembly, SI‐ATRP

## Abstract

Durable and conductive interfaces that enable chronic and high‐resolution recording of neural activity are essential for understanding and treating neurodegenerative disorders. These chronic implants require long‐term stability and small contact areas. Consequently, they are often coated with a blend of conductive polymers and are crosslinked to enhance durability despite the potentially deleterious effect of crosslinking on the mechanical and electrical properties. Here the grafting of the poly(3,4 ethylenedioxythiophene) scaffold, poly(styrenesulfonate)‐*b*‐poly(poly(ethylene glycol) methyl ether methacrylate block copolymer brush to gold, in a controlled and tunable manner, by surface‐initiated atom‐transfer radical polymerization (SI‐ATRP) is described. This “block‐brush” provides high volumetric capacitance (120 F cm^─3^), strong adhesion to the metal (4 h ultrasonication), improved surface hydrophilicity, and stability against 10 000 charge–discharge voltage sweeps on a multiarray neural electrode. In addition, the block‐brush film showed 33% improved stability against current pulsing. This approach can open numerous avenues for exploring specialized polymer brushes for bioelectronics research and application.

## Graphical abstract

Creating durable, conductive interfaces is crucial for studying and treating brain disorders. Here a versatile method is described to graft a block copolymer from gold surfaces, enhancing stability without sacrificing electrical properties. This “block‐brush” coating exhibits high capacitance, strong adhesion, and improved stability against voltage sweeps and current pulsing. This innovation holds promise for long‐term neural stimulation and advances in bioelectronics research.

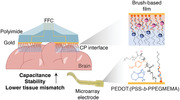

## 1 Introduction

Understanding and treating neurological disorders—originating from injury, aging, and genetics—is highly dependent on the ability to record neural activity in high resolution and over long‐time intervals.^[^ ^1^, ^2^, ^3^ ^]^ Electrocorticography (ECoG) is a technique that measures brain activity from the cortical surface or dura mater and allows both recording and stimulation with high‐resolution.^[^ ^4^, ^5^ ^]^ The basis of this interaction is the detection and manipulation of ionic currents resulting from the action potential of firing neurons at the interface between the electrode and the tissue electrolytes (Figure S1, Supporting Information).^[^ ^6^ ^]^ To achieve such high‐resolution recording of the neural activity, there is a need for microscale electrodes. However, the trade‐off for using such electrodes with small areas of contact is decreased charge injection capacity (CIC), less efficient charge exchange at the interfacial contact between the tissue and the electrode, and increased impedance.^[^ ^7^ ^]^ The interface impedance plays an important role in recording, specifically affecting the baseline noise during recording. Higher impedance will result in higher noise levels, which will reduce the signal‐to‐noise ratio (SNR).^[^ ^8^ ^]^

**Figure 1:**
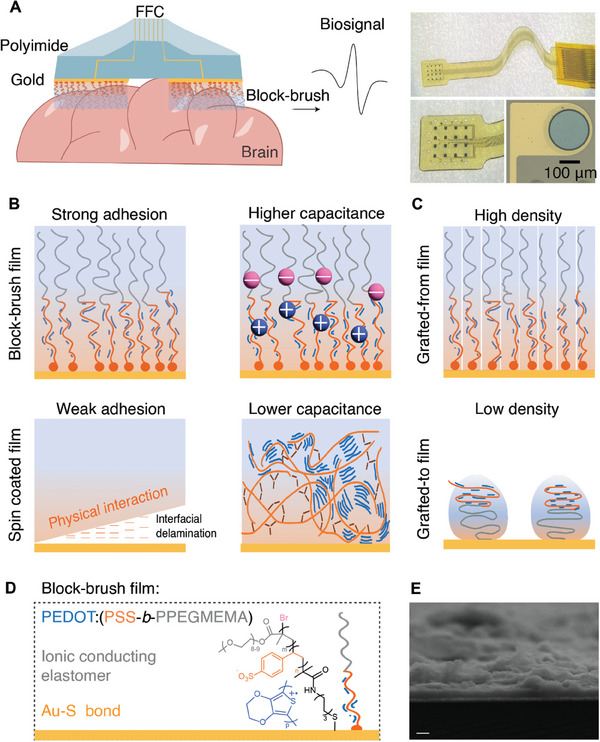
PSS‐*b*‐PPEGMEMA brushes tethered to gold surface as a backbone for PEDOT polymerization enable stable, conductive, conformal, and uniform coverage of the surface. a) Schematic Illustration showing the ECoG microelectrode connected to a flat flex cable (FFC), placed on the brain tissue. Right: The flexible electrode with zoom‐in of the block‐brush tethered to the gold contact's surface. b) The desired properties for the interface between the metal electrode and the brain tissue for long‐term and efficient charge transport during recording or stimulating brain activity, which is facilitated by strong adhesion, electronic and ionic transport, and c) high density of the brushes. The block copolymer brushes PEDOT:PSS‐*b*‐PPEGMEMA, are represented by the following colors, where PSS is orange, PPEGMEMA is gray, and PEDOT is blue. d) Schematic representation and the molecular structure of the PEDOT complexed with PSS‐*b*‐PPEGMEMA brushes, composed of the polyelectrolyte PSS and the ionic conducting elastomer PPEGMEMA. The adhesion to the surface is enabled by the Au‐S bond. e) SEM images of the cross‐section of the gold surfaces. The cross‐section verifies dense, film‐like brushes on the gold surface. The scale bar is 200 nm.

Currently, electrodes for neural interfaces are made of inert metals such as gold and platinum, or metal oxides (e.g., iridium oxide).^[^ ^9^ ^]^ However, films of these inorganic materials are 2D and thus have reduced area for electrochemical interfacing. A common strategy to increase the electrochemical surface area is to coat the metal with a conductive polymer.^[^ ^9^ ^]^ Particularly, polymers capable of mixed ionic and electronic charge transport and with large, 3D electrochemically active surface areas (**Figure** **1**a). These characteristics lead to significantly reduced impedance, and thus a higher (SNR) and CIC, as well as reduced heating of the metal during its operation.^[^ ^10^ ^]^ The biocompatible and commercially available poly(3,4 ethylenedioxythiophene):poly(styrenesulfonate) (PEDOT:PSS) is a prominent electronic‐ionic mixed‐conductive material for coating neural interface.^[^ ^11^ ^]^ The polymeric nature of PEDOT:PSS facilitates the penetration of ions into the matrix, and the electrical conductivity creates an electrical double layer (EDL) throughout the bulk of the film. The EDL and capacitance increase as the coating becomes thicker or rougher.^[^ ^6^ ^]^ Increased surface area correlates with the increased charge storage capacity (CSC) of the electrode^[^ ^12^ ^]^ and may facilitate greater CIC than conventional metallic films.^[^ ^13^ ^]^

In addition to maximizing the capacitance and charge injection, it is also critical to reduce the mechanical mismatch between the polymer and soft biological tissue. This mismatch between conventional metal‐based rigid bioelectronic devices and soft brain tissue can reach several orders of magnitude (hundreds of GPa vs several kPa, respectively).^[^ ^14^ ^]^ This difference in mechanical properties might introduce a mechanical barrier at the interface with the tissue which might result in creating gaps between the tissue and the electrode. Hence, it may limit current injection and potentially induce neural damage and glial scar formation.^[^ ^10^ ^]^ While PEDOT:PSS coating can reduce this mechanical mismatch, reported conventional formulations of PEDOT:PSS have moduli of several hundred MPa to a few GPa.^[^ ^15^, ^16^, ^17^ ^]^ Hence, numerous attempts have aimed to further decrease Young's modulus of the PEDOT‐based conductive coatings using polymer blends,^[^ ^11^ ^]^ hydrogels,^[^ ^18^ ^]^ and PSS chain engineering.^[^ ^19^ ^]^

The durability of conventional formulations of this polymeric coating and strategies to attach these coatings to the metal electrodes (i.e., by van der Waals forces alone) are a concern for implants intended for long‐term use.^[^ ^9^ ^]^ Damage caused by electrochemical reactions at the interface may change the composition and the integrity of the coating over time. In addition, the polymer interface must withstand the corrosive environment of the brain, shear forces with the tissue, and immune responses of the host.^[^ ^20^ ^]^ Moreover, the interactions between the polymer coating and the underlying noble metal electrodes are often weak.^[^ ^21^ ^]^ Therefore, there is a significant incentive to improve the adhesion between the film and the metal, to avoid possible swelling, delamination, and even detachment from the metal surface.^[^ ^22^ ^]^

Robust interfaces require either strong bonds between the polymer and the metal, high interfacial areas, or both.^[^ ^23^ ^]^ Enhancing adhesion can be achieved by increasing the surface area via etching to create pores^[^ ^24^ ^]^ or incorporating a nanostructured rod layer.^[^ ^22^ ^]^ Alternatively, an adhesion layer can be spin‐coated on a surface that is prefunctionalized with amines^[^ ^25^ ^]^ or grown electrochemically directly on the metal.^[^ ^26^ ^]^ These approaches require additional fabrication steps and/or specific types of substrates. Another prominent strategy to enhance the thin film stability is crosslinking the PEDOT:PSS with (3‐glycidyloxypropyl)trimethoxysilane (GOPS). For example, Dijk et al. demonstrated that PEDOT:PSS crosslinked with GOPS, spin‐coated on gold electrodes, remained stable during 4 months of incubation in culture media conditions at 37 °C.^[^ ^27^ ^]^ Despite exhibiting prolonged stability in vitro, the crosslinking of the PEDOT:PSS film with GOPS reduces its electrical conductivity.^[^ ^28^ ^]^ Therefore, additives such as ethylene glycol (EG) and dodecylbenzenesulfonic acid (DBSA) have been used to improve electrical, mechanical,^[^ ^29^, ^30^ ^]^ and wettability of the film.^[^ ^31^ ^]^ However, this approach using additives poses a risk of leaching which can result in device failure and increased toxicity. Other attempts to increase the adhesion include chemical approaches, such as covalently tethering the polymer to the surface. These approaches include either grafting the styrenesulfonate monomers from the surface^[^ ^32^ ^]^ or growing the PEDOT from the surface in a ladder‐like polymer brush.^[^ ^33^ ^]^ These attempts showed a potential for improved stability against sonication and irradiation, respectively. However, the conductivity of the PEDOT‐based brushes was inferior to that of the commercially available material, and the mechanical properties were not evaluated.^[^ ^32^, ^33^ ^]^ Our laboratory recently demonstrated surface‐initiated atom transfer radical polymerization (SI‐ATRP) of PSS, emphasizing the importance of grafting density to create film‐like polymer brushes. Importantly, the PEDOT:PSS polymer brushes showed enhanced stability in comparison with a spin‐coated film.^[^ ^34^ ^]^

Here, we report a strategy that simultaneously addresses three aspects of conductive polymer brush coatings that may limit their widespread adoption in neural recordings: 1) the molecular scaffold in conductive polyelectrolyte complexes has so far been limited to homopolymers such as PSS; 2) as such, mechanical mismatch and conformability at the molecular scale may lead to poor mechanical contact with biological tissue; and 3) low surface hydrophilicity can reduce ionic transport at the interface with biological tissue. We address these issues by using a conductive block copolymer. Previous attempts to use conductive polymer brushes have focused on increasing long‐term stability,^[^ ^33^ ^]^ introducing antifouling properties,^[^ ^35^ ^]^ and creating sites for molecular functionalization for chemical sensing.^[^ ^36^ ^]^ This work emphasizes the use of block copolymer brushes for well‐defined conductive layers for improvement of the surface physical, and electrochemical properties while simultaneously promoting long‐term stability.

## 2 Results and Discussion

### 2.1 Synthesis of Conformal PEDOT‐Based Polymer Brush on Gold Surfaces

Current strategies for surface grafting generally employ nonliving free radical polymerization, which leads to a random assembly of monomers.^[^ ^37^, ^38^ ^]^ However, using living free radical polymerization, such as SI‐ATRP, allows for the creation of a well‐defined multifunctional block copolymer brush with high grafting density (Figure 1b,c and Figures S2–S4, Supporting Information). Hence, we can add functionalities to the polymer using suitable monomers in a bottom‐up approach, without the use of additives. These additives include ethylene glycol or polyethylene glycol derivatives that serve to soften the polymer while also increasing the conductivity.^[^ ^39^, ^40^ ^]^ However, the leaching of additives could result in loss of functionality and introduction of potentially toxic species into tissue. Hence, to allow better conformability with the brain^[^ ^19^ ^]^ we designed and synthesized a second, soft, block copolymer composed of poly(poly(ethylene glycol) methyl ether methacrylate (PPEGMEMA), i.e., PSS‐*b*‐PPEGMEMA (Figure 1d and Figure S3, Supporting Information). Moreover, to improve the stability of the polymer brush against hydrolysis, we used an amide bond between the gold‐bound thiol and the ATRP initiator (Figure S3, Supporting Information). The amide bond demonstrates higher stability and tends to hydrolyze at a slower pace compared to the less stable bonds such as esters.^[^ ^41^ ^]^

**Figure 2:**
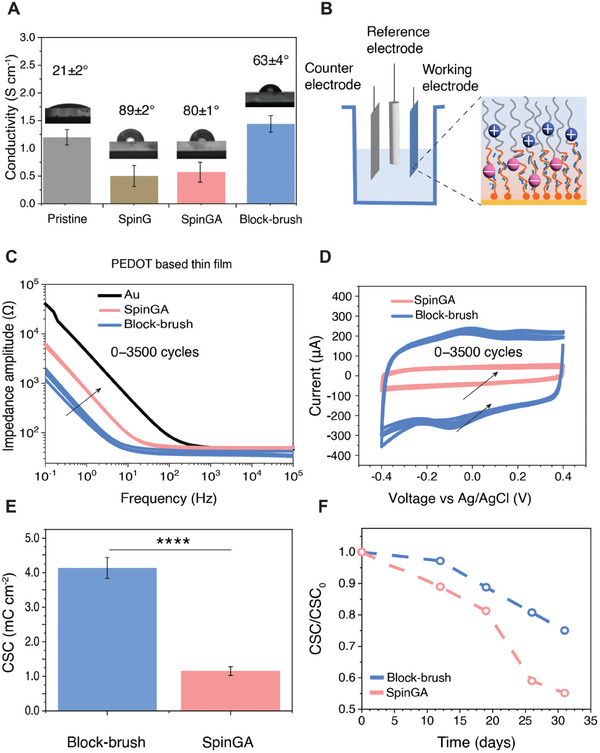
Electrical properties and stability tests of the adhesive films. a) The conductivity of the films including, pristine, SpinG, SpingGA, and block‐brush (*n* = 3). The insets indicate the water contact angle measurements of the different PEDOT‐based films on gold substrates. The block‐brush PEDOT demonstrated the lowest water contact angle among the stable films. We note that the pristine PEDOT:PSS film is not considered a stable film as it dissolves upon contact with water. b) Schematic illustration of EIS/CV three‐electrode setup. The inset shows EDL in the block‐brush polymer film. c) EIS and d) CV curves of bare gold and block‐brush film on gold before and during multiple CV cycling. The block‐brush films are stable during 3500 cycles of CV stressing (0.4 to −0.4 V) e) Characterization of the CSC of the block‐brush film versus the SpinGA film before incubation in PBS (*n* = 3, *P = *2.85 × 10^−6^, 0.8 to −0.4 V). ****P < 0.0001 using one‐tailed *t*‐test. f) The block‐brush films show a slower decrease in CSC over 31 d of incubation in PBS at 50°. The SpinGA films show a faster decrease to 80% of the initial CSC (CSC_0_) only 12 d after incubation and to 55% after 31 d of incubation.

Using SI‐ATRP (“grafting‐from”), we demonstrate high grafting densities with a mechanically stable film of PEDOT:PSS‐*b*‐PPEGMEMA. PEDOT was dispersed in this block copolymer brush via oxidative polymerization in an aqueous phase, resulting in a conductive PEDOT:(PSS‐*b*‐PPEGMEMA brushes) polyelectrolyte complex, i.e., block‐brush film, tethered from the gold surface (Figures S5–S7, Supporting Information). Moreover, a scannig electron mycroscopy (SEM) imaging verified that the polymer brushes formed a dense coating due to their extended conformation (Figure 1e). Importantly, the presence of the hydrophilic PPEGMEMA block led to a decrease in the water contact angle from 80° and 89° for PEDOT:PSS spin‐coated films, crosslinked with GOPS (SpinG) or crosslinked with GOPS and additives (SpinGA), respectively, to 63° (**Figures** **2**a and S8, Supporting Information). Importantly, the block brush had a lower contact angle than the PEDOT:PSS brush, 63° versus 80°, respectively (Figure S8, Supporting Information). This indicates that the addition of the PPEGMEMA block at the upper layer of the surface increases the surface hydrophilicity. Increased hydrophilicity of the conductive polymeric film might increase capillary adhesion to biological tissue and facilitate ion injection into the film.^[^ ^12^ ^]^ To our knowledge, this is the first report of PEDOT:(PSS‐*b*‐PPEGMEMA) polymer brushes grown from a gold surface of a flexible electrode for long‐term ECoG recording of brain activity, with improved charge storage capacity and lower mismatch with the brain.

### 2.2 The Block‐Brush Film Shows Improved Electrochemical Properties

To elucidate the role of the brush morphology on the conductivity and charge storage capacity of the film, we evaluated its electronic and electrochemical properties. As a control, we selected a formulation composed of the commercial PEDOT:PSS crosslinked with GOPS and mixed with EG and DBSA additives, which is widely used for neural recording,^[^ ^27^ ^]^ and also contains polyethylene glycol units (though in the control film they are not bound covalently to the polymer). The block‐brush film had a very similar electrical conductivity to pristine PEDOT:PSS, but were about three times higher than the spin‐coated formulations, SpinG, and SpinGA (GOPS and EG, DBSA additives) (Figure 2a and Figure S7, Supporting Information). Next, we characterized the impedance and capacitive properties of high‐ and low‐density brushes (Figures S9 and S10, Supporting Information). These quantities are important predictors of the quality of recordings achievable. The low‐density block‐brush grafted to the gold surface has a lower electrochemical area than that of the high‐density block‐brush grafted from the gold surface (Figure 1c). Hence, we hypothesized that the EDL of the grafted‐from block‐brush should be higher. As expected, the high‐density brushes, resulted in the lowest impedance below 1 kHz (Figure 2b–d and Figure S10, Supporting Information). This low impedance might be attributed to a larger interfacial capacitance that is also responsible for CSC (Figure 2e), and lower voltage build‐up (Figure S11, Supporting Information). The capacitance per unit area calculated by the standard equivalent circuit was found to be 2200 µF cm^−2^ (Figure S12, Supporting Information).^[^ ^42^ ^]^ The thickness of the film directly influences the volumetric capacitance, which is correlated with the EDL, and the amount of charge that can be injected.^[^ ^6^, ^12^ ^]^ The block‐brush film had a thickness of about 180 nm which is thinner than other reported PEDOT‐based films such as 200 nm − 180 µm^[^ ^43^ ^]^ or 700 nm.^[^ ^44^ ^]^ The latter had a comparable areal capacitance for a film that is 3.8 times thicker (700 vs 180 nm). Normalizing the areal capacitance with the thickness resulted in a volumetric capacitance of 122 F cm^−3^ (Figure S12, Supporting Information). The volumetric electrical double layer capacitance (EDLC) for the block‐brush had a similar value of 107 F cm^−3^ which was six times higher than that of the control SpinGA formulation (Figure S13, Supporting Information).^[^ ^45^ ^]^ This volumetric capacitance is comparable to a previously obtained non‐crosslink stable PEDOT‐based coating that was adhered to the surface using a monolayer of GOPS.^[^ ^46^ ^]^ We further evaluated the electrochemical stability of the block‐brush PEDOT coating during multiple CV` cycles and an accelerated aging test. Importantly, potential sweeps can also harm the film electrochemically, by charge build‐up. The block‐brush films showed higher electrochemical stability for up to 3500 cycles, compared to the crosslinked film that started to delaminate after 2500 cycles (Figure S14, Supporting Information). This is higher than other reported covalently bound dopants with up to 50% loss of the original CSC over only 800 CV cycles.^[^ ^47^ ^]^ The impedance spectra and the area under the curve of the CV for the block‐brush presented negligible changes over the repeating cycles of CV, demonstrating a very high retention of its original CSC (Figure S14, Supporting Information). The optical microscopy inspection revealed that the block‐brush films did not show any damage during 3500 CV cycles, nor for the whole incubation time of 31 d at 50 °C (Figure 2c–f and Figure S15b,f, Supporting Information) while the SpinGA films showed damage starting from day 12 of incubation at 50 °C (Figure S15c,g, Supporting Information). This correlates to 75 and 35 d for block‐brush vs SpinGA films, respectively at a body temperature of 37 °C. Importantly, the CSC of the brushes was more stable with a slower decrease in comparison with the control SpinGA formulation (Figure 2e,f and Figures S15 and S16, Supporting Information).

### 2.3 The Block‐Brush Film Provides Strong Adhesion and Stability of the Bulk Films

**Figure 3:**
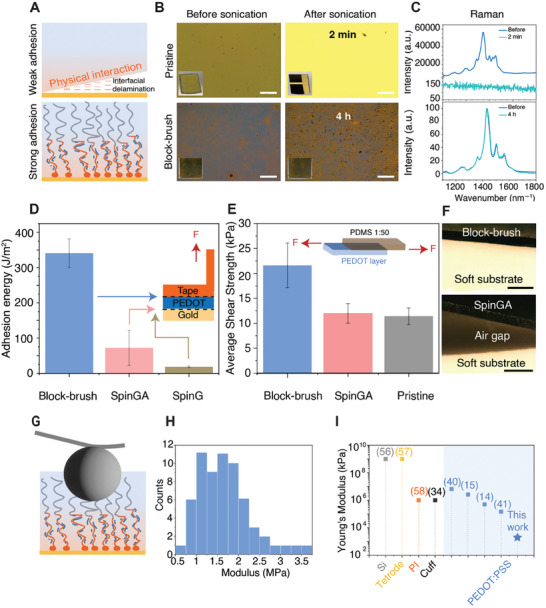
Mechanical stability of the bulk films. a) Schematic illustration of the weak adhesion of spin‐coated pristine PEDOT:PSS versus the strong adhesion of block‐brush PEDOT. b) Corresponding optical microscope images of the films before (left) and after (right) ultrasonication tests. The scale bars are 100 µm. c) Corresponding Raman spectra before (blue) and after (cyan) ultrasonication, show complete PEDOT removal for the pristine sample after 2 min ultrasonication, versus the negligible difference in PEDOT spectrum for the brushes after 4 h sonication. d) 90° Peel (glass/Cr/Au/PEDOT‐based film/PI tape) test for the PEDOT‐based films. e) Average shear strength between PDMS (1:50) and PEDOT‐based films on Au/Cr/glass. Data are shown for pristine (92 ± 3 nm), SpinGA (167 ± 20 nm), brush (115 ± 7 nm), and block‐brush (180 ± 46 nm), (*n* = 3). f) High‐resolution optical images displaying excellent conformability of the block‐brush grown of PDMS on a soft substrate (top). The SpinGA on PDMS does not conform to the soft substrate and presents an air gap (bottom). The scale bar is 0.5 mm. g) Schematic Illustration of the AFM tip indenting block‐brush film for nanomechanical characterization. h) The Young's modulus of 1.7 ± 0.6 MPa (in water) was calculated via the Dimitriadis model, using force deformation curves and a deformation map (Figure S21, Supporting Information). i) Comparison of previously reported conductive materials‐based films with our work in terms of Young's modulus. Such conventional implantable electrical probes include silicon electrodes,^[^ ^77^ ^]^ tetrode,^[^ ^78^ ^]^ planar polyimide probes^[^ ^79^ ^]^ and flexible Au–PET` cuff electrodes^[^ ^43^ ^]^ or PEDOT formulations, which include acid‐treated PEDOT:PSS hydrogel,^[^ ^51^ ^]^ electrodeposited PEDOT,^[^ ^16^, ^17^ ^]^ and spin‐coated PEDOT:PSS with 1% GOPS.^[^ ^52^ ^]^

To test the adhesion between the gold surface and the brushes, we challenged the films with ultrasonication (100 W at 40 kHz) (**Figure** **3**a,b). The block‐brush films remained stable against ultrasonication for up to 4 h, with no detectable damage by optical microscopy (Figure 3b) or Raman spectroscopy (Figure 3c). The low‐density block‐brush film remained on the gold surface; however, they developed cracks within 30 min of ultrasonication, emphasizing the importance of the high density of grafting for a stable film (Figure S17, Supporting Information). The pristine PEDOT:PSS spin‐coated film was fully delaminated after only 2 min of ultrasonication, showing the weakest adhesion to the gold surface. To the best of our knowledge, a stability duration of 4 h represents the longest period ever reported for PEDOT film on metal subjected to ultrasonication. It indicates that the covalent bond of the brushes with the gold surface provides strong adhesion and stability. The stronger adhesion of the brush‐based versus spin‐coated films to the gold surface was also demonstrated by the higher force needed for a 90° peel‐off test. We found that for the block‐brush films the delamination occurs at the brush/tape interface while for the SpinG and SpinGA films, it occurs at the PEDOT film/Au interface. The block‐brush films were not delaminated following the peel test thanks to the strong Au‐S bonds. The adhesion energy was improved from 18.15 and 72 J m^−2^ for SpinG and SpinGA respectively to 341.05 J m^−2^ for the block‐brush (Figure 3d and Figure S18, Supporting Information). A comparable adhesion force was obtained previously for the delamination of films made of random hydrogels bound to the surface of electrodes.^[^ ^38^, ^48^ ^]^ Furthermore, we postulated that the rougher surface of the brush‐based films (Figure S12, Supporting Information) would translate to a higher adhesion against soft surfaces compared to the spin‐coated films. Indeed, the lap‐joint shear strength against soft polydimethylsiloxane (PDMS) (with a ratio of 1:50)^[^ ^49^ ^]^ for the brush‐based films was higher compared to the spin‐coated PEDOT films (Figure 3e and Figure S19, Supporting Information). We further evaluated the conformability of the brushes by placing the block‐brush film on a soft substrate. The block‐brush demonstrated excellent conformability to the surface, with no air gaps, compared to the SpinGA sample which did not adhere well to the soft surface (Figure 3f).

### 2.4 Nanomechanical Characterization

Elevated levels of PEDOT relative to PSS are linked with enhanced conductivity. However, this advantage is counterbalanced by an accompanying increase in modulus, leading to a material with greater stiffness.^[^ ^50^ ^]^ Hence, we investigated the nanomechanical properties of block‐brush film in liquid, to mimic the wet environment of the brain (Figure S20, Supporting Information). Using nanoindentation of the nanometer‐thick film, we quantified the mechanical response of the polymer to the force applied by the AFM` tip (Figure 3f and Figures S7, S20, and S21, Supporting Information). The resulting Young's modulus for the soft stretchable block‐brush film was 1.7 ± 0.6 MPa (Figure 3h). This Young's modulus is lower than other reported electrodes for neural recording materials or PEDOT formulations,^[^ ^16^, ^17^, ^51^, ^52^ ^]^ approaching that of the brain (dura mater) tissue (several MPa) (Figure 3i).^[^ ^53^ ^]^

### 2.5 Block‐Brush on Thin‐Film Microelectrode Arrays

**Figure 4:**
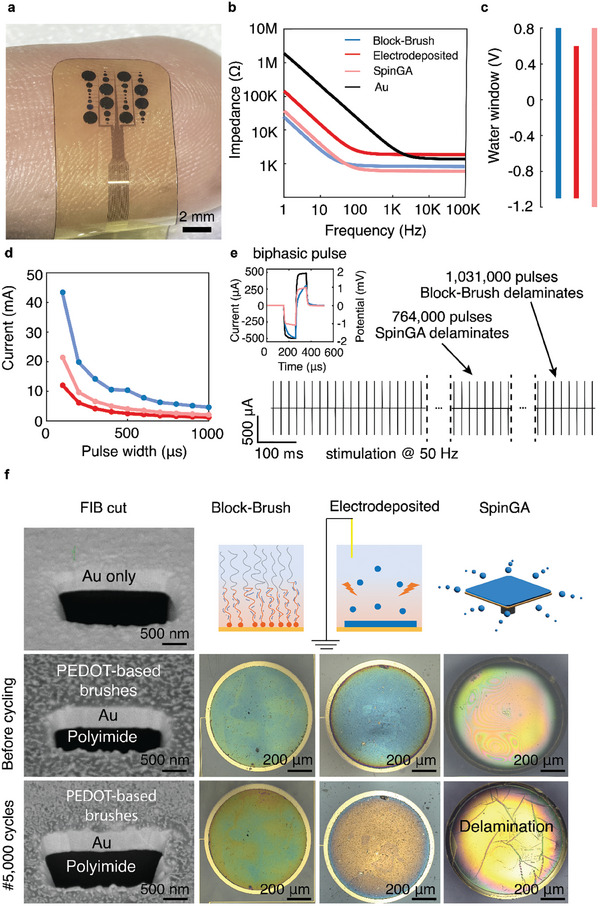
Electrochemical characterization of multidiameter microelectrode array and films long‐term stability. a) Image of the 32‐channel electrode array containing 100, 200, 400, and 1000 µm diameter contacts. b) Impedance spectrum and c) water window comparison between the three PEDOT‐based thin films for the 1000 µm diameter contacts. d) CIC for the three PEDOT‐based thin films for the 400 µm diameter contact. e) Breakdown of the 400 µm electrode contacts during biphasic current pulse stressing. Pulses were delivered at 50 Hz. Top left inset: Example biphasic pulse delivered during current stressing. f) Left: Focused ion beam (FIB) image of the block‐brush contact before and after 5000 CV cycles, showing no change to the film morphology. Right: Microscope images of the 1000 µm diameter contacts before (top) and after (bottom) 5000 CV cycles.

To evaluate the block‐brush film as an efficient and stable interface for high‐resolution neural interfaces, we fabricated a polyimide‐based thin‐film electrode with microsized gold contacts of varying diameters from 100 µm to 1 mm (**Figure** **4**a and Figures S22–S25, Supporting Information). This range spans the contact sizes typically used in microelectrode arrays for neural applications.^[^ ^8^ ^]^ To demonstrate the versatility of our strategy, the PEDOT was electrodeposited on the PSS‐*b*‐PPEGMEMA polymer brushes. As an additional control that is commonly used in bioelectronics, PEDOT:PSS was spin‐coated on the microelectrode array that was fabricated by us (Figure S26a, Supporting Information). The spin‐coated control showed some disadvantages in comparison with the brushes. First, the fabrication process is longer (Figure S26a, Supporting Information) and requires an additional peel‐off step to pattern the PEDOT:PSS selectively on the gold contacts (Figure S26b, Supporting Information). Second, there is a greater chance for damage at the edges of the contacts (Figure S26c, Supporting Information). The block‐brush PEDOT electrodes showed a similar EIS spectrum and water window to the control SpinGA and electrodeposited samples (Figure 4b and Figures S27 and S28, Supporting Information). Importantly, CIC was highest for the brush‐based coating across all pulse widths and all diameters (Figure 4d and Figure S29, Supporting Information). This difference in CIC is significant and is higher than previously reported PEDOT‐based films.^[^ ^54^ ^]^ Moreover, the CSC was highest for the block‐brush film in comparison with control formulations (Figure S30, Supporting Information), which was consistent with the results from the bulk film. These results suggested that the brush‐based PEDOT:PSS can deliver higher stimulation amplitudes and may be more stable during long‐term, repeated current injection. To validate this supposition, we stressed the block‐brush film and the SpinGA formulation with biphasic current pulsing. We delivered 500 µA amplitude and 100 µs duration cathodic‐first biphasic pulses at 50 Hz to a 400 µm contact. These parameters are within the current amplitude and frequency range of typical neural stimulation.^[^ ^55^, ^56^ ^]^ The contact with the SpinGA control formulation delaminated after around 750 000 pulses, while the contact with the block‐brush delaminated after around 1 000 000 pulses, constituting a 33% improvement in stability (Figure 4e). These results highlight the improved long‐term stability during stimulation of the brush‐based PEDOT films. Next, we evaluated the long‐term stability of the PEDOT‐based coatings using CV cycle stressing. We found that the block‐brush coating was highly stable against 5000 voltage sweeps, while the SpinGA formulation showed multiple regions of delamination under optical microscopy (Figure 4f). Moreover, all materials showed stable impedance spectra even up to 10 000 cycles (Figure S31, Supporting Information), which stands in line with former reports of IrOx,^[^ ^57^ ^]^ Au nanorods,^[^ ^22^ ^]^ and GOPS^[^ ^46^ ^]^ adhesion promoters.

### 2.6 In Vivo Implantation and Neural Recording

**Figure 5:**
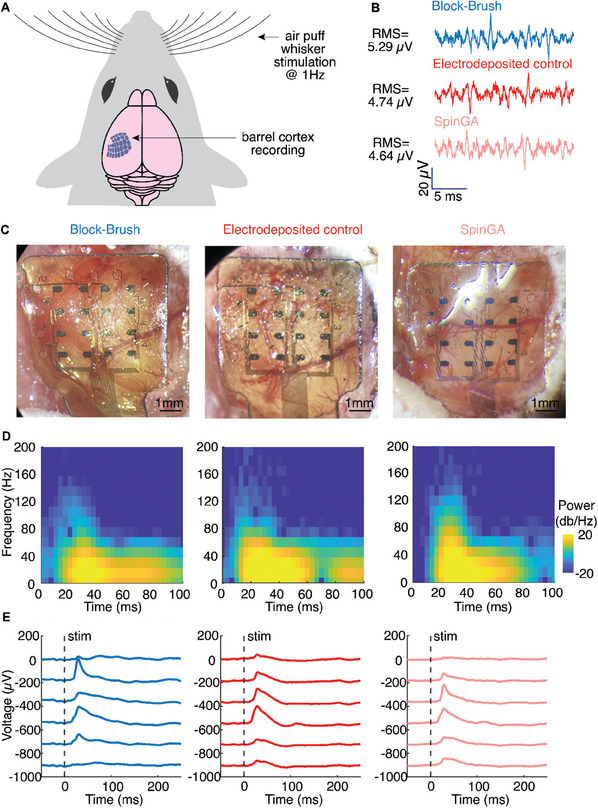
Block‐brush PEDOT film on microelectrode array record somatotopic functional cortical columns. (a) Schematic of the rat brain implanted with a 16‐channel, 4.8 mm‐by‐4.8 mm array, and the air puff stimulation of individual whiskers. b) Baseline recording of the brain activity, with baseline RMS values for the three materials. c) Magnified microscope image of the electrode on the rat barrel cortex. d) Spectral analysis of the mean trial‐averaged response across low‐impedance channels to whisker air puff stimulation for the block‐brush (left), electrodeposited control (middle), and SpinGA control (right) samples. Responses from the three materials showed similar spectral profiles, with onset time ≈20 ms poststimulus and high power in frequency range < 80 Hz. e) Trial‐averaged responses from six individual low‐impedance channels from each array, block‐brush (left), electrodeposited control (middle), and SpinGA control (right). The dashed line indicates the time of air puff stimulation. Responses from the three materials are similar in shape and amplitude, indicating that the block‐brush film can capture neural activity.

To validate the neural recording capabilities of the block‐brush coating, we recorded the whisker barrel activity in anesthetized rats subjected to repeated air‐puff stimulation of the whiskers (**Figure** **5**a). The rat barrel cortex exhibits a well‐defined organization of somatosensory cortical structures that map one‐to‐one with the whiskers.^[^ ^58^ ^]^ We designed a 16‐channel array with 200 µm contact diameters coated with block‐brush and control formulations (Figure 5c). We first measured baseline noise recorded by the microelectrode arrays and found the root mean square (RMS) noise to be similar between the three formulations (Figure 5b). The neural activity recorded with the block‐brush electrode showed a similar spectral response, with activity recorded at a range of frequencies up to high gamma (<190 Hz) (Figure 5d and Figure S32, Supporting Information). The raw waveforms exhibited a similar shape across materials with peak responses observed ≈25 ms after the onset of the air puff (Figure 5e). The block‐brush electrode exhibited a similar peak‐to‐peak amplitude response and SNR compared to the SpinGA and the electrodeposited controls (Figure S33, Supporting Information). In summary, these results confirm that the brush‐based PEDOT film can capture all relevant frequency components of neural activity similar to a spin‐coated formulation of PEDOT:PSS. Importantly, the block‐brush approach enhanced stability was evidenced through in vitro and in vivo stress testing, including aging, voltage swipes, current injection, and mechanical testing. Hence it may be an essential coating for neural interfaces needed for chronic use.

## 3 Conclusions

The generation of stable interfaces for recording and stimulation of neural activity holds promise for research and clinical applications requiring chronic implantations. Here, we demonstrate for the first time the SI‐ATRP of block‐brush PEDOT with a full characterization of nanomechanical, electrical, electrochemical, and long‐term stability properties together with a successful recording of neural activity. This chemical pathway is compatible with a polyimide‐based electrode, resulting in a uniform, conductive, and stable film. Moreover, the SI‐ATRP living polymerization nature enables us to design and synthesize block copolymers with combined functionalities in a film that is composed of one component. This is advantageous to other common strategies such as blending two different polymers, which are prone to phase segregation and loss of function over time.

Some studies have demonstrated a lower CSC and CIC for PEDOT:PSS coatings in comparison with other coating materials, such as PtNR.^[^ ^59^ ^]^ This study can open new directions for exploring novel avenues to enhance the electrochemical properties of PEDOT:PSS coating through the modulation of polymer brush segments, density, or composition. Our results showed that the brush‐based film had superior electrical properties in comparison with the commonly used approach in which a spin‐coated film is crosslinked and was on par with coating materials with high CIC, such as PtNR. The improvement in electrochemical properties can be attributed to the open pathways within the extended polymer brushes network and the greater level of ordering at the molecular level.^[^ ^60^ ^]^ This morphology might facilitate greater charge movement than the crosslinked formulation.^[^ ^61^, ^62^ ^]^ Moreover, the enhancement in electrochemical properties, achieved without the need for additional dopants or treatments, hints at the potential for implementing the block‐brush in various applications requiring both biocompatibility and good conductivity. This potential extends to areas such as the development of conductive scaffolds, particularly in situations where high density of PEDOT is crucial. An added advantage is no restriction to specific types of substrates as was demonstrated before for surface‐initiated polymerization from PDMS,^[^ ^63^ ^]^ Silicon,^[^ ^64^ ^]^ and polyesters.^[^ ^65^ ^]^ Other relevant areas necessitate avoiding stringent conditions such as acid treatment^[^ ^66^ ^]^ or thermal processes (e.g., annealing),^[^ ^67^ ^]^ which are incompatible with living cells or tissues.

## 4 Experimental Section

### General

Number‐average molecular weight (*M*_n_), weight‐average molecular weight (*M*_w_), and dispersity (*D̵*) were determined using an Agilent Technologies 1260 Infinity II LC system. The mobile phase was 30% methanol and 70% 0.2 m NaNO_3_, and 0.01 m NaH_2_PO_4_ in water at pH 7 (adjusted with concentrated NaOH) at 40 °C at 1 mL min^–1^. The PL aquagel‐OH Mixed‐B column was used, calibrated against narrow dispersity PSS standards (purchased from Polymer Standards Service). ^1^H NMR spectra were acquired in D_2_O at room temperature on a Bruker AVANCE III 600 MHz NMR spectrometer fitted with a 1.7 mm triple resonance probe with the z‐gradient

### Materials

Sodium 4‐styrenesulfonate (NaSS), 4,4′‐azobis(4‐cyanovaleric acid) (ACVA), azobis(isobutyronitrile), poly(ethylene glycol) methyl ether acrylate (PEGMEA, *M*_n_ = 480 g mol^–1^), 4‐cyano‐4‐(phenylcarbonothioylthio)pentanoic acid (the reversible addition‐fragmentation transfer (RAFT) chain transfer agent), 6‐Amino‐1‐hexanethiol hydrochloride, Cu(I) bromide, Cu(II) bromide, 2,2′‐bipyridine, ethyl α‐bromoisobutyrate, sodium chloride, poly(ethylene glycol) methyl ether methacrylate (PPEGMEMA, *M*_n_ = 500 g mol^–1^), and ethylenedioxythiophene (EDOT) were purchased from Sigma‐Aldrich and used without further purification. A commercially available formulation of PEDOT:PSS (Clevios PH1000, Heraeus) as well as EG dopants, and crosslinker GOPS and stabilizer dodecylbenzenesulfonic acid (DBSA) were purchased from Sigma‐Aldrich and used without further purification. Triethylamine and methanol were obtained from Fisher Scientific and used without further purification. Distilled water filtered using a Milli‐Q purification system was used throughout.

**(I) Synthesis of PEDOT: (PSS‐*b*‐PPEGMEMA) Brushes on Gold (Grafting‐to and Grafting‐from)**

It was aimed to compare two strategies of binding the polymers to the gold surface, grafting‐to, and grafting‐from. It was hypothesized that the grafting‐from approach would provide a more uniform coverage of the gold surface, with a stretched regime of the polymer brushes (Figure S2, Supporting Information).

1) Grafting‐to: By RAFT Polymerization of PSS_(1)_‐*b*‐PPEGMEA`_(6)_ Block Copolymer

PSS_(1)_‐*b*‐PPEGMEA_(6)_ was synthesized as previously described.^[^ ^19^ ^]^ Briefly, PSS macro‐RAFT was synthesized by RAFT polymerization of sodium styrene sulfonate (NaSS) monomers. The RAFT agent was 4‐cyano‐4‐(phenylcarbonothioylthio)pentanoic acid, and the initiator was (4,4′‐Azobis(4‐cyanovaleric acid)) ACVA. The reaction ratio was 0.2:1:150 initiator:RAFT agent:monomers. The reaction was stopped by exposure to air. PSS macro‐RAFT was purified by precipitation in acetone and dried under vacuum to afford a pink powder. The molecular weight of the PSS was determined by gel permeation chromatography (GPC): *M*w = 31.2 kDa, *Ð* = 1.3. Next, to synthesize the PSS‐*b*‐PPEGMEA, PSS macro‐RAFT, the ACVA initiator and PEGMEA monomers were polymerized via second RAFT polymerization. The reaction ratio was 0.2:1:400 initiator:PSS macro‐RAFT agent:monomers. The ^1^H NMR of the crude mixtures showed 93% PEGMEA conversion. The molecular weight of the PSS_(1)_‐*b*‐PPEGMEA_(6)_ was determined by GPC: *M*w = 100 kDa, *Ð* = 1.74.

2) Grafting‐from of Poly(sodium 4‐styrenesulfonate) (PSS) by SI‐ATRP

The polymerization of sodium styrene sulfonated was carried out as previously described.^[^ ^34^ ^]^ Gold‐coated Si wafers (100 nm Au, 10 nm Cr adhesion layer, Si) were cleaned by sonicating in Alconox, acetone, and 2‐propanol for 10 min each. The sample was dried with compressed air and then oxygen plasma treated for 10 min before soaking in 1 mM 6‐Amino‐1‐hexanethiol hydrochloride in ethanol for 24 h. The resulting 6‐Amino‐1‐hexanethiol hydrochloride‐coated gold was rinsed with ethanol and then transferred to a new flask. The flask was purged and refilled with nitrogen (x3) before adding anhydrous tetrahydrofuran (THF) and triethylamine (1.1 eqv). α‐Bromoisobutyryl bromide (0.1 m, 1 eqv) was added and the solution was gently stirred under nitrogen for 3 min before the samples were removed and rinsed with ethanol and DI` water. The sample was dried under compressed air and used immediately for the polymerization of PSS.

SI‐ATRP grafting from PDMS (Sylgard 184 Silicone) was performed similarly to a previously described procedure.^[^ ^63^ ^]^ PDMS was mixed in a 10:1 base‐to‐curing agent ratio and degassed under vacuum to remove air bubbles. PDMS was spun‐coated on top of a glass substrate at 500 rpm (250 rpm s^─1^) for 60 s. The substrate was cured at 70 °C in an oven for 8 h. After curing, the substrate was treated with oxygen (O_2_) plasma for 3 min and (3‐Aminopropyl)trimethoxysilane was drop cast on the PDMS and heated at 80 °C for 5 min. The resulting (3‐ Aminopropyl)trimethoxysilane‐coated PDMS was rinsed with ethanol and then transferred to a new flask. The next steps were identical to the 6‐amino‐1‐hexanethiol hydrochloride‐coated gold surface modification.

The initiator‐coated gold surface was added to a clean vial. A stock solution of the polymerization reactants was prepared in a separate flask under nitrogen. Sodium styrene sulfonate (NaSS) (4.12 g, 1000 eq), copper (I) bromide (0.0029 g, 1 eq), copper (II) bromide (0.0015 g, 0.33 eq), 2,2′‐bipyridine (0.0050 g, 1.6 eq) were added to a round bottom flask and purged and refilled (x3). NaCl (0.2338 g, 200 eq) was added before purging. A 3:2 mixture of MilliQ water and methanol was sparged with nitrogen for at least 2 h to remove all oxygen. 31 mL of sparged DI/methanol was added to the stock solution (0.65 m NaSS). The solution was stirred vigorously to dissolve all reagents and turned a light tan. Once all reagents were solubilized, ethyl α‐bromoisobutyrate (eBiB) (2.9 µL, 1 eq) was added to initiate the synthesis of free polymer. Immediately after adding eBiB, 5–8 mL of the stock solution was transferred into the flask containing the gold sample under nitrogen to begin SI‐ATRP. The reaction was terminated by opening the flask to air. The gold surfaces were rinsed with DI water (Figure S3, Supporting Information).

3) Grafting‐from of Poly(sodium 4‐styrenesulfonate‐block‐poly(poly) (PSS‐b‐PPEGMEMA) by SI‐ATRP

Due to the living nature of radical polymerization, we can tailor the molecular structure by adding a second, soft, block copolymer to decrease the mechanical mismatch with the tissue and the hydrophobicity of the PEDOT film. The polymerization of the second block of PPEGMEMA was modified from Robinson et al.^[^ ^68^ ^]^ The PSS‐modified gold surface was added to a clean vial. A stock solution of the polymerization reactants was prepared in a separate flask under nitrogen. Poly(ethylene glycol) methyl ether methacrylate (PPEGMEMA) (9.663 g, 1000 eq), copper (I) bromide (0.0029 g, 1 eq), copper (II) bromide (0.0015 g, 0.33 eq), 2,2′‐bipyridine (0.0050 g, 1.6 eq) were added to a round bottom flask and purged and refilled (x3). A 3:2 mixture of MilliQ water and methanol was sparged with nitrogen for at least 2 h to remove all oxygen. 31 mL of sparged DI/methanol was added to the stock solution (0.65 m NaSS). The solution was stirred vigorously to dissolve all reagents and turned a light tan. Once all reagents were solubilized, ethyl α‐bromoisobutyrate (eBiB) (2.9 µL, 1 eq) was added to initiate the synthesis of free polymer. Immediately after adding eBiB, 5–8 mL of the stock solution was transferred into the flask containing the gold sample under nitrogen to begin SI‐ATRP. The reaction was terminated by opening the flask to air. The gold surfaces were rinsed with DI water (Figure S3, Supporting Information).

4) Oxidative Polymerization of EDOT on PSS‐b‐PPEGMEMA Brushes

PSS‐*b*‐PPEGMEMA brushes on gold were used as a scaffold for the polymerization of EDOT following literature procedures.^[^ ^32^ ^]^ Briefly, the PSS‐*b*‐PPEGMEMA brushes were immersed in 0.1 m aqueous solution of EDOT and stirred vigorously in a sealed vial for at least 15 h before adding FeCl_3_ • 6H_2_O (0.75 m). Blue particles formed over the reaction time (12–48 h). Once the PEDOT polymerization was complete, the sample had a bluish tint and was rinsed with DI water and dried in a desiccator overnight (Figure S3, Supporting Information).

5) Grafting to Gold Surfaces of Poly(sodium 4‐styrenesulfonate‐block‐poly(poly) (PEDOT:PSS‐b‐PPEGMEA)

First, the macro‐RAFT agent block copolymer, PEDOT:PSS_(1)_‐*b*‐PPEGMEA_(6)_ was reduced to the thiol‐exposed polymer by a procedure modified from Kayser et al.^[^ ^69^ ^]^ Briefly, PEDOT:PSS‐*b*‐PPEGMEA (4485 mg, 4.152 µmol, 25 mL) was mixed with 2‐ethanolamine (19.6 µL, 200 eq.) and tributylphosphine (150 µL, 18 eq.) was then added using a syringe. The reaction mixture was left to stir for 18 h at room temperature (Figure S4, Supporting Information). Then, to the 25 mL aqueous polymer dispersion of Thiol‐ended PEDOT:(PSS‐*b*‐PPEGMEMA‐SH) 3196 mg of Na_2_SO_4_ was added, to create a final concentration of 0.9 m Na_2_SO_4_, according to modified procedures from literature.^[^ ^70^, ^71^ ^]^ The Na_2_SO_4_ salt was added to increase the grafting density during the formation of polymer brush layers.^[^ ^70^ ^]^ In addition, to ensure the thiol is reduced during the grafting process, 2.8 mL of 100 mM TCEP` was also added to the mixture, to create a final concentration of 10 mM. The mixture was vigorously stirred for 3 h. Finally, the PEDOT:(PSS‐*b*‐PPEGMEA‐SH) was grafted to the gold surface. Gold‐coated Si wafers (100 nm Au, 10 nm Cr adhesion layer, Si) were cleaned by sonicating in Alconox, acetone, and 2‐propanol for 10 min each. The sample was dried with compressed air and then oxygen plasma treated for 10 min before soaking in the reduced PEDOT:(PSS‐*b*‐PPEGMEA‐SH) solution. The grafting process was kept stirring at ambient for 3 d. At the end of the incubation, the surfaces were vigorously washed with DI water, to remove unbound polymer, and kept at ambient.

**(II) Chemical Characterization**

Further analysis of chemical composition was done using a Nicolet iS50 Fourier transform infrared spectrometer (Figure S5, Supporting Information). Raman spectroscopy was performed on Renishaw inVia upright microscope using a 532 nm source. SEM micrographs were captured on a Zeiss Sigma 500 SEM with an accelerating voltage of 3.00 kV and an InLens detector. Chemical composition was confirmed via X‐ray photoelectron spectroscopy (XPS) performed at UC Irvine Center for Complex and Active Materials on a Kratos AXIS‐Supra. A survey was acquired at low x‐ray intensity (5 mA) and detailed elemental spectra were acquired at 20 eV and 40 eV at 20 mA (Figure S6, Supporting Information). The thickness of PEDOT:PSS and PSS films was measured using SEM and ellipsometry on a J.A. Wollam M‐2000D spectroscopic ellipsometer (Figure S7, Supporting Information) with a beam size of 3 mm. Surface profilometry measurements were also conducted on the DektakXT Stylus Profiler to obtain the film thicknesses. All the measurements were taken with a vertical range of 6.5 µm and a Stylus force of 3 mg. Five measurements were taken of each sample to obtain the average thickness. Film thickness was estimated assuming a 5 nm chromium layer and a 100 nm gold layer on silicon. Water contact angle images were obtained with a ramé hart Model 200 goniometer (Figure S8, Supporting Information).

**(III) Electrical and Electrochemical Characterization**

1) PEDOT Film Deposition

The working electrodes were deposited/grafted on glass or Si substrates. The glass/Si substrates (2.5 cm×2.5 cm) were cleaned sequentially by 10 min of sonication cycles in soap water, DI water, acetone, and isopropanol. Before use, the cleaned glass substrates were treated by a UV Ozone reactor for 10 min at 30 W and 450 mTorr. 10 nm Cr adhesion layer and 100 nm Au layer were then deposited by thermal evaporation using Orion System, AJA` International. Next, the slide was cleaned sequentially by 10 min sonication with ethanol and DI water. The thin films grafted‐to or grafted‐from the surface were created as described above. The spin‐coated films were fabricated as follows. Before spin coating of PEDOT ink, the Au‐coated slides were treated by a UV Ozone reactor for 10 min at 30 W and 450 mTorr. Spin‐coated films were prepared as described previously.^[^ ^72^ ^]^ Briefly, PEDOT:PSS (Clevios) was mixed with 1% GOPS (SpinG). 20 mL aqueous dispersion of PEDOT:PSS was mixed with 5 mL EG, 50 µL of DBSA, and 1% wt% GOPS (SpinGA). PEDOT:PSS without additional materials is referred to as Pristine PEDOT:PSS. The different solutions were spun‐coated on top of Glass/Cr/Au or Si/Cr/Au substrates at 500 rpm (250 rpm s^─1^) for 120 s, followed by 2000 rpm (1000 rpm s^─1^) for 10 s. Following deposition, films were annealed at 120 °C for 15 min on a hot plate under ambient atmosphere before being allowed to slowly cool down to room temperature by removing them from the hot plate.

2) Electrical Conductivity

The resistances of the films were measured using a four‐point probe wired to a Keithley Standard Series 2400 Source Measure Unit, with a probe spacing of 2 mm and a sample size of 25 × 25 mm^2^. The thickness of the films was measured using a Dektak XT profilometer or by ellipsometry, and the cross‐sectional area was used to convert resistance to conductivity. The conductivity, *σ*, was calculated from an average of three samples using the following equations:

(1) $$R_{s}=\frac{\pi }{\ln\left(2\right)}\;R\;$$

(2) $$\sigma =\frac{1}{t\times R_{s}}\;\;$$

where *R* is the resistance measured by the four‐point probe, *R*_s_ is the sheet resistance, and *t* is the thin film thickness. A correction factor of 0.9497 was applied according to the geometry of the measurement.

3) Electrochemical Impedance Spectroscopy and Cyclic Voltammetry Measurements

The EIS and CV measurements were used to evaluate the electrochemical activity of our different PEDOT‐containing films. We first compared the two grafting methods, grafting‐to, and grafting‐from (Figure S9, Supporting Information). Later the grafting‐from films against the spin‐coated formulations (Figure S1, Supporting Information) were compared. Gamry interface 1000E was used to perform EIS in 0.01 m 1 × PBS` solution (consisting of 0.022 m Na_2_HPO_4_ (pH = 7.2 ± 0.2), using a three‐electrode configuration, i.e., PEDOT electrodes as the working electrodes, Ag/AgCl electrode as a reference electrode, a platinum rod electrode as a counter electrode. 10 mV root‐mean‐square (RMS) sinusoidal signal with zero DC bias was applied and the frequency was swept from 0.1 Hz to 1 × 10^6^ Hz. The capacitance and electrical double layer testing were measured by cyclic voltammetry under low current density, near equilibrium conditions in (1×) PBS solution, with the tested electrode potential swept cyclically within the potential windows of −0.4–0.8 V or −0.4–0.4 V relative to the Ag/AgCl electrode at a constant scan rate of 100 mV s^−1^ with 5 mV potential steps. The current was injected in biphasic, anodic, and cathodic stimulation, from 1 mA up to 36 mA, with a voltage limit of ± 10 V (Figure S11, Supporting Information). All voltammetric measurements were conducted in an ambient atmosphere (Figure S12, Supporting Information). Finally, five different scan rates were studied (20, 40, 60, 80, and 100 mV s^−1^) (Figure S13, Supporting Information).

The specific capacitance of the film was obtained according to Equation (3):

(3) $$C*=\frac{\oint jdV}{2\upsilon \left(V_{\mathrm{ma}x}-V_{\min}\right)*A}\;\;\;\;\;$$

where integration is performed over the area of the voltammogram, *j* is the current density, *V* is the voltage, *υ* is the scan rate, and *A* is the area of the film.

The volumetric capacitance of the film was calculated according to Equation (4):

(4) $$C*=\frac{\oint jdV}{2\upsilon \left(V_{\max}-V_{\min}\right)*v}\;\;$$

where integration is performed over the area of the voltammogram, *j* is the current density, *V* is the voltage, *υ* is the scan rate, and *v* is the volume of the film. Calculations are summarized in Figures S12 and S13 in the Supporting Information.

**(IV) Mechanical Stability Characterization**

1. Accelerated aging test: PEDOT‐based films, including the block‐brush, and SpinGA were challenged by an accelerated aging test in PBS, pH 7.4, at 50 °C for 35 d. EIS and CV measurements and optical microscope inspections were taken every several days as described above. The size of the exposed areas was 6 mm × 8 mm (Figure S14, Supporting Information).
2. Oxidative reactive accelerated aging test: PEDOT‐based films, including the brushes, SpinG (0.2% wt% GOPS), and the brushes grafted to the gold surface, were incubated in 20 mM H_2_O_2_ in PBS at 50 °C for 56 d (Figure S15, Supporting Information). This procedure was modified from literature.^[^ ^73^ ^]^
3. Ultrasonication stability test: The PEDOT‐based film, including the block‐brush, grafted from and to the gold surface, and Pristine PEDOT:PSS were challenged by ultrasonication (100 W at 42 kHz) for different time points (Figure S16, Supporting Information).
4. Peel test: To conduct a 90° peel test, a Mark‐10 linear actuator equipped with the peel test accessory kit was operated in the upright position. Samples were taped with Kapton tape for 12 h before the measurements. The glass/Cr/Au/PEDOT films were fixed to the sliding plate using double‐sided tape. The edge of the Kapton tape was attached to a grip connected to a 10 N force gauge. The tapes were removed at a rate of 330 mm min^–1^ to obtain a plot of force relative to displacement (travel) (Figure S17, Supporting Information). The adhesion force between the surface of the films and the PDMS (1:50), is calculated from the max force achieved during the shear test divided by the joint area of the films. The adhesion energy is calculated from the force at the plateau divided by the sample width (2.5 cm).
5. Adhesion force between the PEDOT films and soft PDMS surface: PDMS (Sylgard 184 Silicone) was mixed in a 50:1 base‐to‐curing agent ratio and degassed under vacuum to remove air bubbles. PDMS was then deposited using a plastic cup on a glass slide and spun at 1000 rpm (500 rpm s^─1^) for 60 s. The substrate was cured at 70 °C in an oven for 3 h. Lap‐joint shear tests (shear properties) were performed against the soft 50:1 PDMS, at a rate of 1.4 mm min^−1^ (Figure S18, Supporting Information).
6. Electrical stability: The stress testing was performed in terms of cyclic voltammetry under low current density, near equilibrium conditions in (1×) PBS solution, with the tested electrode potential swept cyclically within the potential windows of −0.6–0.4 V relative to the Ag/AgCl electrode at a constant scan rate of 100 mV s^−1^ with 5 mV potential steps (Figure S19, Supporting Information).

**(V) AFM Characterization**

AFM measurements were carried out with a Bruker Innova. Topographical imaging was done with tapping mode, 160AC NA tips from MikroMasch were used in air and qp‐BioAC tips produced by Nanosensors were used for liquid imaging in DI water. Mechanical measurements were conducted in DI water with Biosphere B500‐CONT tips made by Nanotools and indentation curves were fit to the Dimitriadis model using custom MATLAB scripts. The thickness of the brush for the model fitting was found by scraping away an area of brush in air with an HQ:NSC14/Hard/Al BS tip, leaving behind the bare gold substrate, washing, and measuring the height of the neighboring brushes in tapping mode after equilibration in water for 1 h. The indentation and retraction rates were 100 nm ^−1^s and 100 points were chosen for indentation near the area where height was measured. The deflection sensitivity was found by calibration on a silicon substrate after cleaning in piranha solution for 30 min and copious rinsing in DI water.

Elastic modulus characterization of the block‐brush film. The elastic deformation was obtained by analysis of AFM‐tip‐polymer brush interactions by nanoindentation, according to the Dimitriadis model,^[^ ^74^, ^75^ ^]^ using Equation (5).

(5) $$\begin{array}{ccc} F & = & \frac{4E}{3\left(1-\nu^{2}\right)}\;R^{1/2}\delta^{3/2}\left[1-\frac{2\alpha_{0}}{\pi }\chi +\frac{4{\alpha_{0}}^{2}}{\pi^{2}}\chi^{2}\right.\\ & & \;\left.-\frac{8}{\pi^{3}}\left({\alpha_{0}}^{3}+\frac{4\pi^{2}}{15}\beta_{0}\right)\chi^{3}+\frac{16\alpha_{0}}{\pi^{4}}\left({\alpha_{0}}^{3}+\frac{3\pi^{2}}{5}\beta_{0}\right)\chi^{4}\right]\;\\ \chi & = & \frac{\sqrt{R\delta }}{h_{\mathrm{compressible}}}\\ \alpha_{0} & = & -\frac{1.2876-1.4678\nu +1.3442\nu^{2}}{1-\nu }\\ \beta_{0} & = & -\frac{0.6387-1.0277\nu +1.5164\nu^{2}}{1-\nu } \end{array}$$

*δ* is the deformation due to the force imposed by a spherical indenter, *R* is the tip radius (500 nm), E* is the composite modulus, *ν*_s_ is Poisson's ratio of the substrate (Au, 0.42), *ν*_f_ is Poisson's ratio of the brushes’ “film” (PEG, 0.3) (Figure S21a, Supporting Information).^[^ ^76^ ^]^

**(VI) Microelectrode Array Fabrication**

1) Design and Fabrication of the Multiarray Neural Electrode

The microelectrode arrays were fabricated on 75 × 25 mm^2^ glass slides (Corning), which were cleaned with ethanol and IPA` and baked at 180 °C for 10 min before use. The glass slide was coated with 10‐µm‐thick‐polyimide (PI) 2611 and baked in a carbolite oven (Carbolite Gero) at 350 °C for 1 h. Next, the metal lead traces (10 nm Cr, 150 nm Au) were deposited onto the glass slide using standard lithography techniques and AZ5214E‐IR photoresist (MicroChemicals). A 3 µm PI encapsulation layer was spin‐coated onto the surface and baked at 350 °C for 1 h. Finally, the via holes were patterned with AZ12XT‐20‐10 photoresist (MicroChemicals) and oxygen plasma etched for 30 min. The samples were then soaked in Remover PG` at 80 °C for 1 h to remove photoresist residues. PSS and PSS‐*b*‐PPEGMEMA brushes were then grafted from the exposed microscale Au contact surface.

2) Electrodeposition of PEDOT on the Microelectrode Array

Once PSS brush synthesis was completed, PEDOT was electrodeposited onto the surface of the microscale contacts. The electrodeposition solution was prepared by dissolving 50 µL of 3 4‐ethylenedioxythiophene (EDOT) in 47 mL of DI water. The solution was vortexed until EDOT dissolved completely. Potentiostatic electrodeposition was performed using the Reference 6000 (Gamry Instruments) at 1.1 V for 80  s.

3) Charge Storage Capacity (CSC) Calculation

All reported charge storage capacities were calculated from cyclic voltammograms between −0.6 and 0.6 V. CSC was calculated using Equation (6).

(6) $$\mathrm{CSC}=\sum_{\Delta t\;\in \;T}I\left(t\right)\;\times \;\Delta t/A\;$$

where Δ*t* is a single timestep in the total duration of one CV cycle *T*, *I*(*t*) is the current measured at time *t* in the CV cycle, and *A* is the area of the sample.

4) Charge Injection Capacity (CIC) Calculation

The charge injection capacity for a given contact was calculated with the Reference 6000 (Gamry Instruments) from the water window and the maximum negative polarization potential (*E*_mc_) computed across a range of pulse widths between 100 and 1000 µs and current amplitudes between 3 µA and 10 mA, depending on the contact size and impedance. The CIC was identified as the point of intersection between *E*_mc_ and the negative water window limit.

5) Biphasic Pulse Stimulation

Repeated current pulsing was performed by delivering a train of biphasic, cathodic‐first current pulses with 100 µm pulse width and 500 µA current amplitude. Pulses were delivered at 50 Hz using the RHS` Stim/Recording System (Intan Technologies). The contact was considered delaminated when it could no longer deliver the desired current amplitude, which was identified by a voltage compliance flag on the RHS System.

**(VII) In Vivo Recording of Neural Activity**

1) Vertebrate Animal Subjects

An adult (>5 months old, weight 450 g) male Sprague‐Dawley rat (Charles River Laboratories) was used as the vertebrate animal subject in this study. All animal experiments were approved by the UC San Diego Institution Animal Care and Use Committee (protocol S16020).

2) Surgical Procedures

Rats were sedated with 4% isoflurane and fixed in a stereotaxic frame (Kopf Instruments). Once stable, anesthesia was reduced to 1.5–2.5% during the surgery. A craniotomy was performed, exposing the whisker barrel cortex without breaching the dura. The electrode array was placed onto the whisker barrel cortex. Gel foam (SURGIFOAM Absorbable Gelatin Sponge) was used to cover the microelectrode array to secure it in place and maintain moisture on the brain.

Once the surgery was completed, rats were transitioned from isoflurane to ketamine/xylazine (90 and 10 mg k^−1^ g^−1^, respectively; MWI`) and redosed 20–30 min for the duration of the experiment. Heart rate, body temperature, and blood oxygenation were continuously monitored throughout the experiment. A heating pad maintained body temperature between 34 and 36 °C throughout the experiment. At the end of the study, animals were euthanized with 120 mg k^−1^ g^−1^ sodium pentobarbital (MWI).

3) Data Collection

Once the electrode was in place, the reference needle electrode was placed near the neck of the rat, and ground was connected to the stereotaxic frame. An individual whisker was stimulated with the air puff using the Pneumatic PicoPump (WPI, PV830). Neural activity was recorded with the RHS Stim/Recording System (Intan Technologies). After a 1 min baseline recording, each whisker was stimulated for 2 min at 1 Hz for a total of 120 trials. The air puff stimulation was time locked to the recording system by sending TTL` signals to both the air puff stimulator and the RHS.

4) Data Analysis

A 1‐min fragment of baseline recording (without air puff stimulation) was high pass filtered >300 Hz with a 2nd order Butterworth filter. The RMS of the baseline noise was calculated in MATLAB.

To perform spectral analysis of the whisker barrel cortex activity in response to stimulation, neural recordings were first notch filtered at 60 Hz, then bandpass filtered 0.1–300 Hz using a 2nd order Butterworth filter. The spectral analysis of the trial averaged data (120 trials) was performed in MATLAB using the spectrogram function.

The peak‐to‐peak amplitude of the response was calculated by subtracting the trial averaged minimum potential from the maximum potential over the period between 1 and 200 ms after stimulation. The signal to noise ratio was calculated by dividing the peak‐to‐peak amplitude by the RMS of the baseline noise.

**(VIII) Statistical Analysis**

To normalize the presented data in Figure 2F (aging results), each representative CSC value for 0, 12, 19, 26, and 31 d was normalized to its initial value, as measured before initiating the aging experiment using Matlab software. Figure 2E and Figure S33 in the Supporting Information show the averaged data (mean) and the corresponding SD` was presented in the form of (mean ± SD) and sample size of (*n* = 3). The statistical analysis was carried out using one‐sided *t*‐test. The statistical significance of the peak‐to‐peak was evaluated by the Mann‐Whitney *U* test, using Matlab (ranksum function).

## Conflict of Interest

S.A.D. has competing interests that are not related to this work, including equity in Cortical Sciences Inc. which concerns the commercialization of brain recording and stimulation electrodes, and is a paid consultant to MaXentric Technologies. The other authors declare that they have no competing interests.

## Author Contributions

R.B. and S.M.R. contributed equally to this work. The author contribution is as follows: conceptualization (R.B., S.M.R., S.A.D., D.J.L.); methodology (R.B., S.M.R., Y.Q., W.S., A.L., A.X.C., A.N., L.A., A.A., G.L.E., S.J.E., R.V., S.P.D., M.H., J.V.J., D.P.F., A.R.T., S.A.D., D.J.L.); investigation (R.B., S.M.R., Y.Q., W.S., A.L., L.A.); visualization (R.B., S.M.R., Y.Q., W.S.); funding acquisition (R.B., D.J.L.); project administration (R.B., S.M.R., S.A.D., D.J.L.); supervision (D.J.L., S.A.D.); writing—original draft (R.B., S.M.R.); writing—review and editing (R.B., S.M.R., A.X.C., S.A.D., D.J.L.).

## Supporting information

Supporting Information

## Data Availability

The data that support the findings of this study are available from the corresponding author upon reasonable request.
