# Supplementary material for: Surface‐Grafted Biocompatible Polymer Conductors for Stable and Compliant Electrodes for Brain Interfaces
Source: Adv Healthc Mater. 2024 Jul 16;13(29):2402215. doi: 10.1002/adhm.202402215 (PMC11582513; doi:10.1002/adhm.202402215)
Supplement: Supplementary file 1 — Supporting Information [file ADHM-13-0-s001.pdf]

ADVANCED HEALTHCARE MATERIALS

Supporting Information

for *Adv. Healthcare Mater.*, DOI 10.1002/adhm.202402215

Surface-Grafted Biocompatible Polymer Conductors for Stable and Compliant Electrodes for Brain Interfaces

*Rachel Blau, Samantha M. Russman, Yi Qie, Wade Shipley, Allison Lim, Alexander X. Chen, Audithya Nyayachavadi, Louis Ah, Abdulhameed Abdal, Guillermo L. Esparza, Samuel J. Edmunds, Ritwik Vatsyayan, Sean P. Dunfield, Moumita Halder, Jesse V. Jokerst, David P. Fenning, Andrea R. Tao, Shadi A. Dayeh and Darren J. Lipomi**

Supporting Information for:

Surface-Grafted Biocompatible Polymer Conductors for Stable and Compliant Electrodes for Brain Interfaces

Rachel Blau[1]†, Samantha M. Russman[3]†, Yi Qie[1], Wade Shipley[2], Allison Lim[1], Alexander X. Chen[1], Audithya Nyayachavadi[1], Louis Ah[1], Abdulhameed Abdal[5], Guillermo L. Esparza[1], Samuel J. Edmunds[4], Ritwik Vatsyayan[4], Sean P. Dunfield[1], Moumita Halder[1], Jesse V. Jokerst[1], David P. Fenning[1], Andrea R. Tao[1,2], Shadi A. Dayeh[3,4], Darren J. Lipomi[1]*

Affiliations:

[1]Aiiso Yufeng Li Family Department of Chemical and Nano Engineering, University of California; San Diego, 9500 Gilman Drive, Mail Code 0448, La Jolla, CA 92093-0448, USA.

[2]Materials Science and Engineering Program, University of California, San Diego, 9500 Gilman Drive, Mail Code 0418, La Jolla, CA 92093-0418, USA.

[3]Department of Bioengineering, University of California; San Diego, 9500 Gilman Drive, Mail Code 0448, La Jolla, CA 92093-0448, USA.

[4]Department of Electrical and Computer Engineering, University of California; San Diego, 9500 Gilman Drive, Mail Code 0448, La Jolla, CA 92093-0448, USA

[5]Department of Mechanical and Aerospace Engineering, University of California; San Diego, 9500 Gilman Drive, Mail Code 0448, La Jolla, CA 92093-0448, USA.

*Corresponding author. Email: dlipomi@ucsd.edu

†These authors contributed equally to this work

The PDF file includes:

Figures. S1 to S33

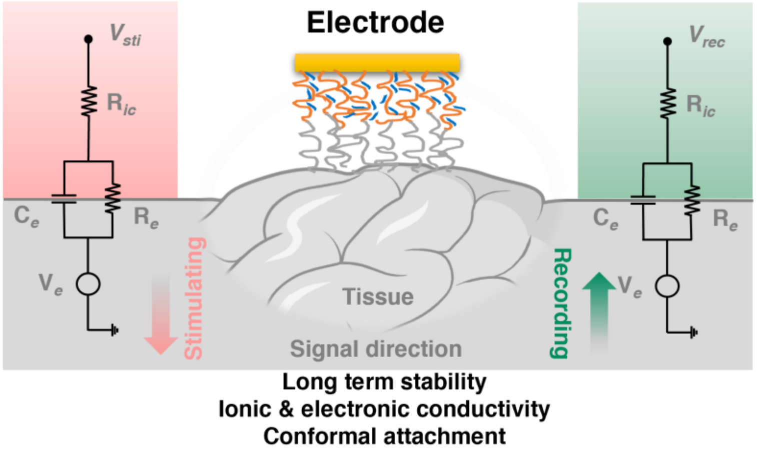

Figure S1. Schematic Illustration showing the ECoG microelectrode placed on the brain tissue. On the sides is the equivalent circuit model of tissue–electrode interfaces for bioelectronic stimulation. V_{sti} represents the input for bioelectronic stimulation with the interconnect resistance R_{ic}. Ve represents the electric potential within electrolytic media generated by V_{sti} applied on the outer membrane of the targeted neurons. The equivalent circuit model of tissue–sinterfaces for bioelectronic recording is on the sides. Below are the desired properties for the interface between the metal electrode and the brain tissue for efficient charge transport during recording or stimulating brain activity.

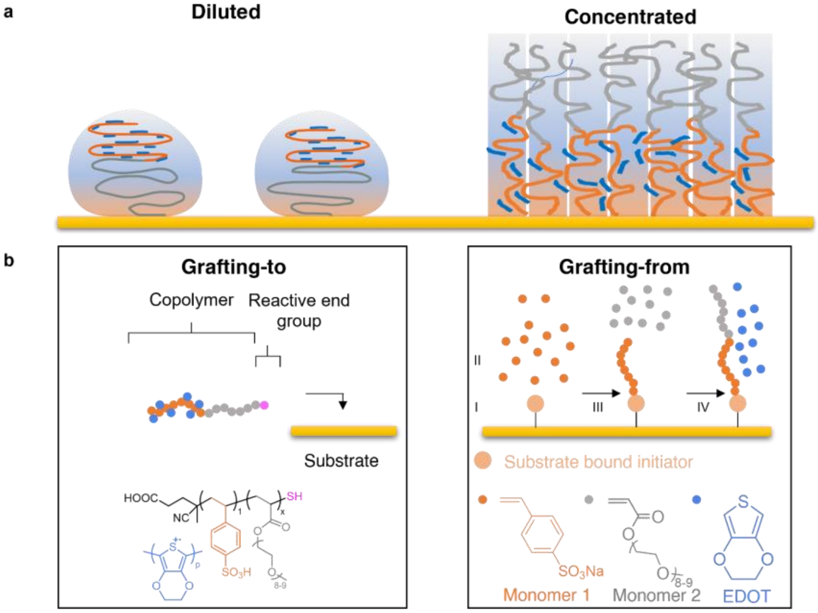

Figure S2. Grafting-from approach forms a high-density, stretched brush regime. (**a**) Illustrations of diluted (low density) and concentrated (high density) block-brushes. (**b**) Corresponding synthesis pathways schemes of grafting-to approach (left) vs. grafting-from approach. The dense regime for the latter is facilitated *via* a reduced steric hindrance of the low molecular weight monomers that are grown from the surface (steps I to IV are detailed in **Figure S3**). For the grafting-to approach, the bulk polymer chain hinders the interaction of additional chains with the surface, resulting in a diluted brush morphology.

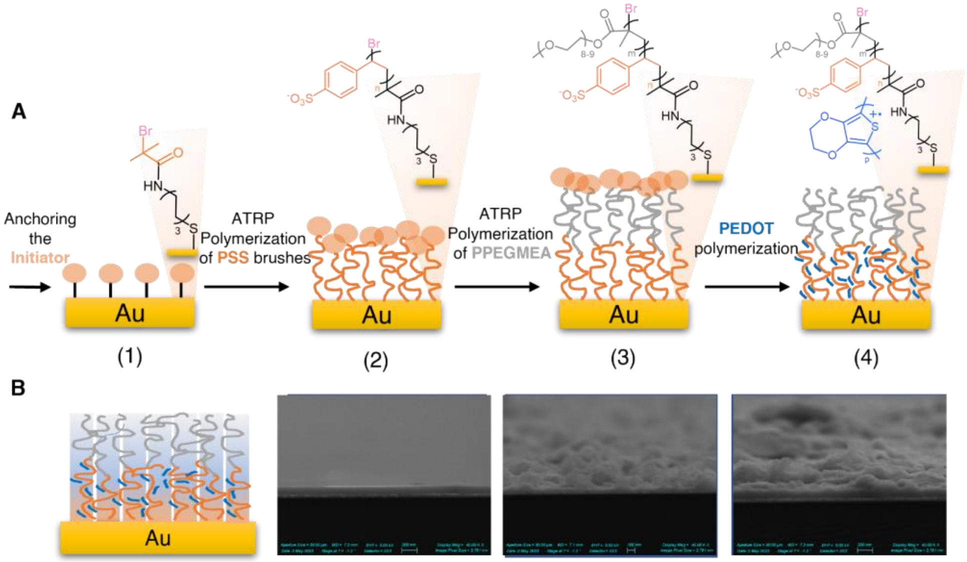

Figure S3. PSS-*b*-PPEGMEMA polymer brushes tethered to the gold surface as a backbone for PEDOT polymerization. (**a**) Gold surface modification with immobilized atom transfer radical polymerization (ATRP) initiator (1). ATRP polymerization of sodium styrene sulfonate (NaPSS, orange) to yield NaPSS covalently bound to Au. The reaction is conducted in H_2O/EtOH, 70°C, N_2 (g) 24 h (2). The second block, PPEGMEMA (gray) is added, with similar reaction conditions as 2 (3). Oxidative polymerization of PEDOT:PSS with $FeCl_3$, Sodium persulphate in Milli-Q water, r.t., o.n (4). The contact angle measurements for each step indicate the degree of hydrophilicity (n=3). Schematic illustration of the stretched brushes obtained by ATRP. SEM images of the cross-section of the gold surfaces corresponding to the synthesis steps on panel (**b**). Illustrations of concentrated (high-density) PEDOT:(PSS-*b*-PPEGMEMA) (block-brush) and the corresponding SEM cross-sections of each synthesis step described in (A), verify dense, film-like, block-brush on the gold surface. The scale bars are 200 µm. Panel (4) is reproduced from Figure 1E to clearly show the different surfaces corresponding to the steps of surface modification.

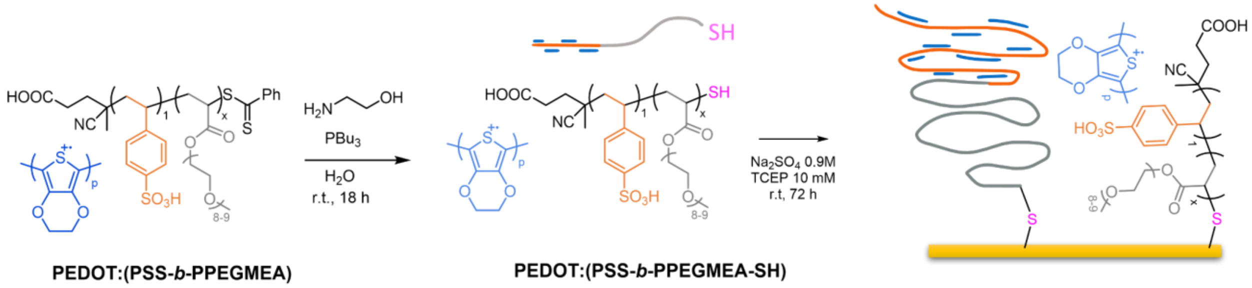

Figure S4. Synthesis scheme of the block copolymer grafted to the gold surface. First, the thiocarbonylthio end group was removed from RAFT-Synthesized Polymers by aminolysis. Later, the conductive block copolymer was bound to the gold surface *via* the exposed thiol (SH) group, in the presence of a high concentration of Na_2SO_4 salt, and Tris (2-carboxyethyl) phosphine (TCEP) reducing agent.

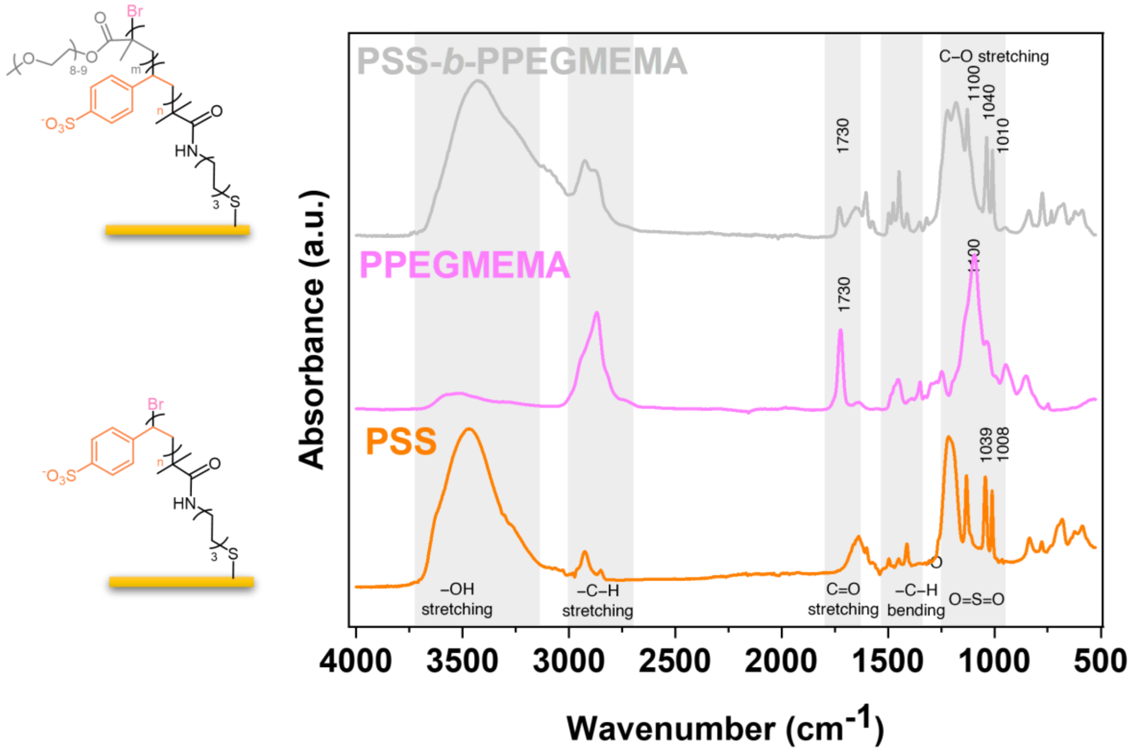

Figure S5. Fourier-transform infrared (FTIR) spectroscopy. PSS brushes (orange) spectrum shows the presence of the following peaks: broad peak around 3440 cm^{-1} (OH stretching), 3064, 3029 cm^{-1} (aromatic C─H stretching), 2923 and 2850 cm^{-1} (asymmetric and symmetric CH2 vibrations), 1176 cm^{-1} (asymmetric SO_2 stretching), 1008, 1039 cm^{-1} (symmetric SO_2 stretch), and 673 cm^{-1} (S─OH).[80,81] PPEGMEMA polymer spectrum (pink) shows the following indicative peaks: a broad peak around 3560 cm^{-1} (OH stretching), 2870 (C─H stretching), 1730 (C=O stretching of the ester group), 1250, and 1290 (CH_2 twisting) 1100 (C─O, C─C stretching).[82,83] The spectrum of block copolymer PSS-*b*-PPEGMEMA brushes (gray).

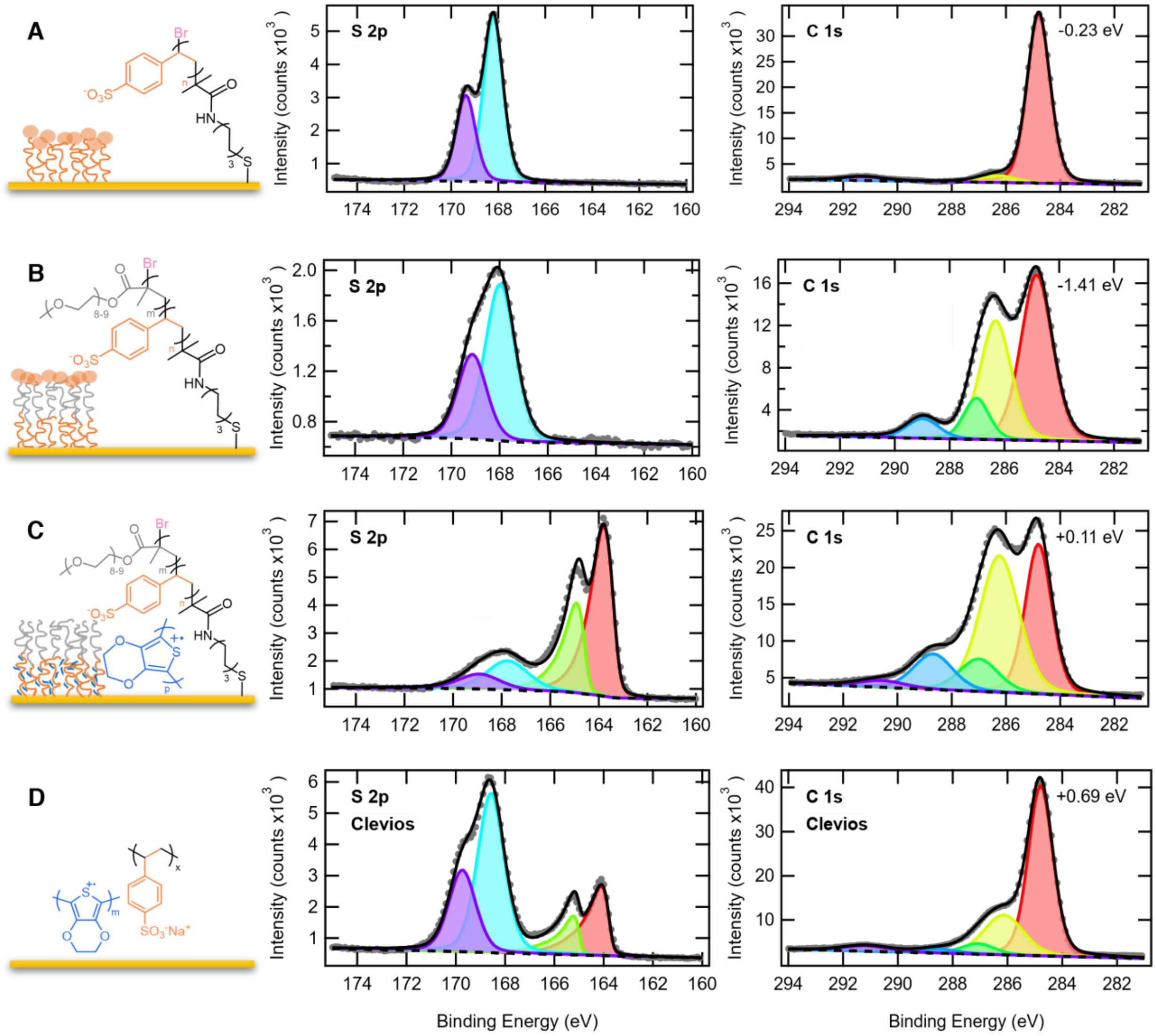

E

	PSS	PEDOT
Peaks	**S 2p**	**S 2p**
	SO_3	C−S−C
Binding Energy [eV]	166-171	163-166
Ratio (n)		
Block-brush	1	3.3
Clevios	3	1

Figure S6. Quantitative analysis of the brushes by X-ray photoelectron spectroscopy (XPS). (**a**) PSS, (**b**) PSS-*b*-PPEGMEMA (**c**) Block-brush (**d**), and Clevios (pristine PEDOT:PSS). The left panel shows the S(2p) peaks of sulfur atoms in the PEDOT chains that revealed two peaks between 167 and 162 eV, while the S(2p) peaks of the PSS chains could be found in the range of 171 to 166 eV[84] The C(1s) peak s of the corresponding carbon atom spectra are on the right panel.

(**c**) For PSS-*b*-PPEGMEMA, the peaks of the different carbon atoms containing PPEGMEMA are indicated, including carbonyl, C=O, groups at 289.2 and 287.3 eV, C−O at 286.6 eV, and C−C/C−H at 285 eV. These peaks are in agreement with previous reports.[85] (**d**) Typical carbon spectra C(1s) of PEDOT:PSS include the aromatic C=C of PEDOT at 284 eV, the aliphatic C−C of PSS chains at 285 eV 13, the C−O/C−S bonds at 286.7 eV, and the C=O/C=S bonds at 290.5 eV.[85–87] (**e**) Quantification of the PEDOT and PSS and ratios according to the XPS peaks, indicating a higher ratio for the block-brush.

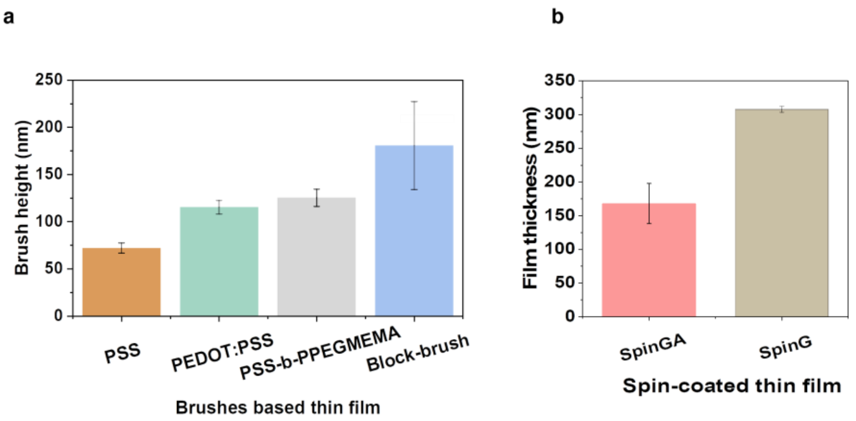

Figure S7. Polymer brush film thickness. The films made of polymer brushes were evaluated by ellipsometry for each step of the synthesis, including (**a**) PSS, PSS-*b*-PPEGMEMA, PEDOT:PSS based brush and PEDOT:PSS-*b*-PPEGMEMA (block-brush). (**b**) Spin-coated PEDOT:PSS film with additives (SpinGA, 1% GOPS, EG, DBSA) and spin-coated PEDOT:PSS film with 1% GOPS (SpinG).

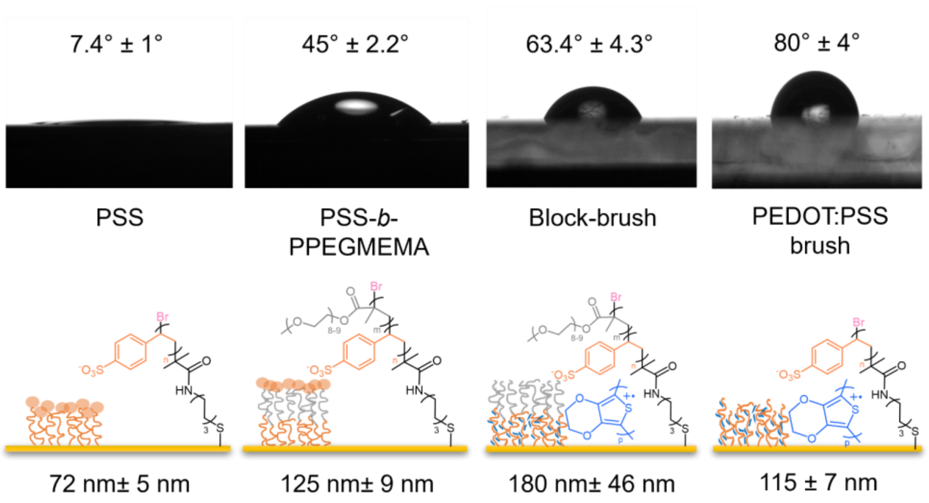

Figure S8. Water contact angle measurements. PSS brushes, PSS-*b*-PPEGMEMA brushes, block-brush and PEDOT:PSS brushes. The number below indicates the thickness of the films, measured by ellipsometry as shown in **Figure S7**. The below 10º water contact angle of PSS indicated a highly hydrophilic surface post PSS SI-ATRP modification. The water contact angle of the PEDOT:PSS-*b*-PPEGMEMA brush (block-brush) is lower than that of PEDOT:PSS brush, probably due to the addition of hydrophilic PPEGMEMA groups to the upper surface of the film.

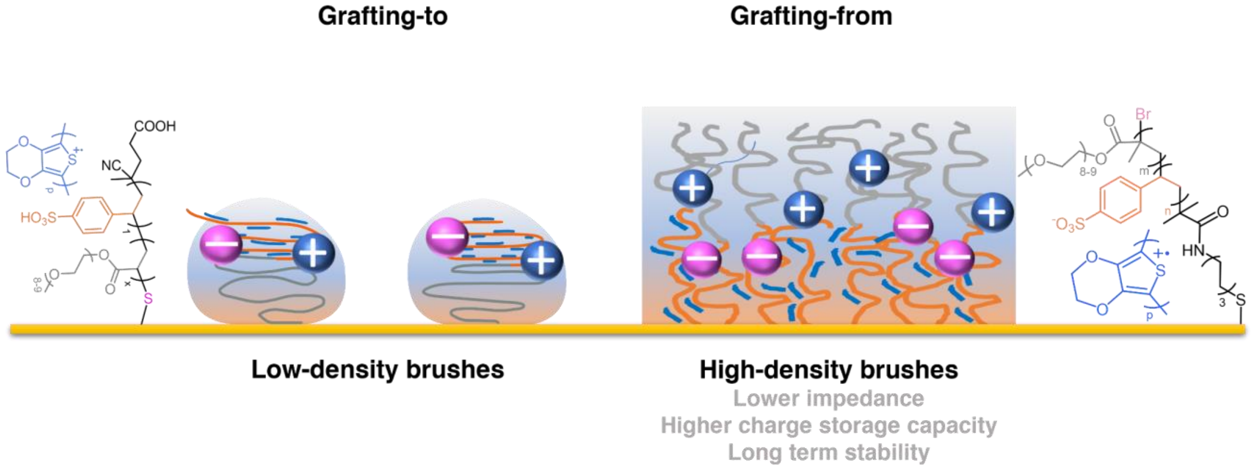

Figure S9. Schematic illustration of the electrical double layer (EDL) in a high-density versus low-density film. Left: low-density block-brush grafted to the gold surface has a lower electrochemical area than that of the high-density block-brush grafted from the gold surface (right). Hence, we hypothesized that the EDL of the grafted-from block-brush should be higher.

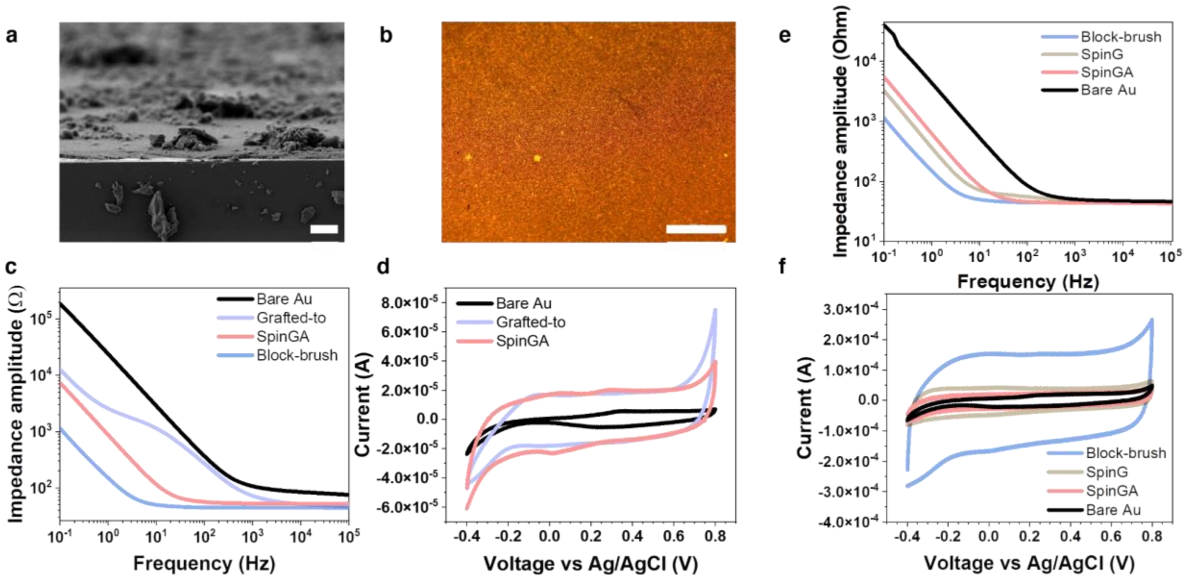

Figure S10. Electrochemical characterization of the PEDOT-based film. (**a**) SEM and (**b**) optical microscope images of block-brush grafted to the gold surface. The scale bars are 2 µm and 500 µm, respectively. (**c**) EIS and (**d**) CV curves of the block-brush grafted to the gold surface in comparison with the bare gold, and SpinGA sample. (**e**) The block-brush film that was grafted from the surface shows the lowest impedance EIS, and (**f**) the highest AUC of the CV compared to bare gold, and spin-coated films on gold.

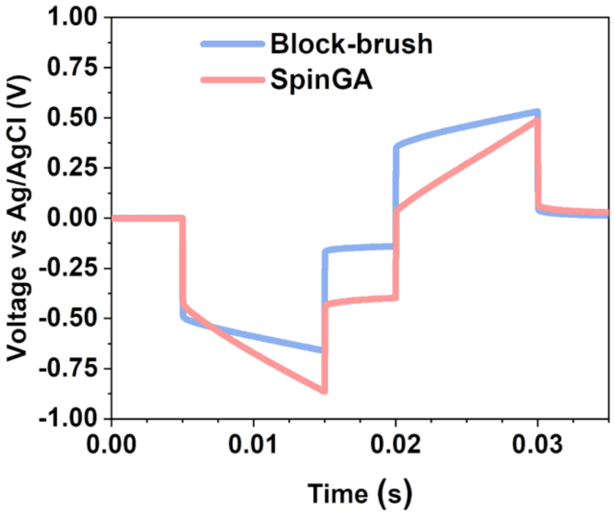

Figure S11. Voltage transient of injected current pulses. The current was injected with a 10 ms pulse width, measured at anodal/cathodal limits for SpinGA and the block-brush.

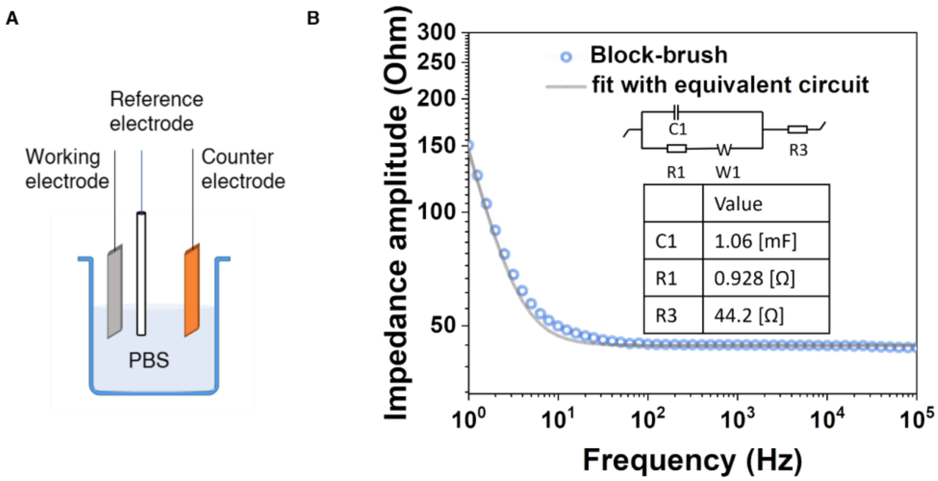

Figure S12. Impedance spectrum and estimation of areal capacitance. (**a**) Schematic illustration of EIS three-electrode setup, immersed in PBS. (**b**) Bode Plot (blue circle) shows impedance versus frequency for block-brush with an electrode area of 0. 48 cm^2 (0.06 cm x 0.08 cm). Inset: the equivalent circuit and the estimated value of its components. C1 is the double-layer capacitance. R1 and R3 are charge transfer resistance and electrolyte resistance respectively. W1 is the Warburg element. The grey line was fit with an equivalent circuit of (R1+W1)//C1+R3. A high capacitance of 2200 $\mu F\ cm^{-2}$ was calculated by dividing C1 by the electrode geometric area. Dividing this value with the thickness of the sample resulted in a volumetric capacitance of 122 $F\ cm^{-3}$.

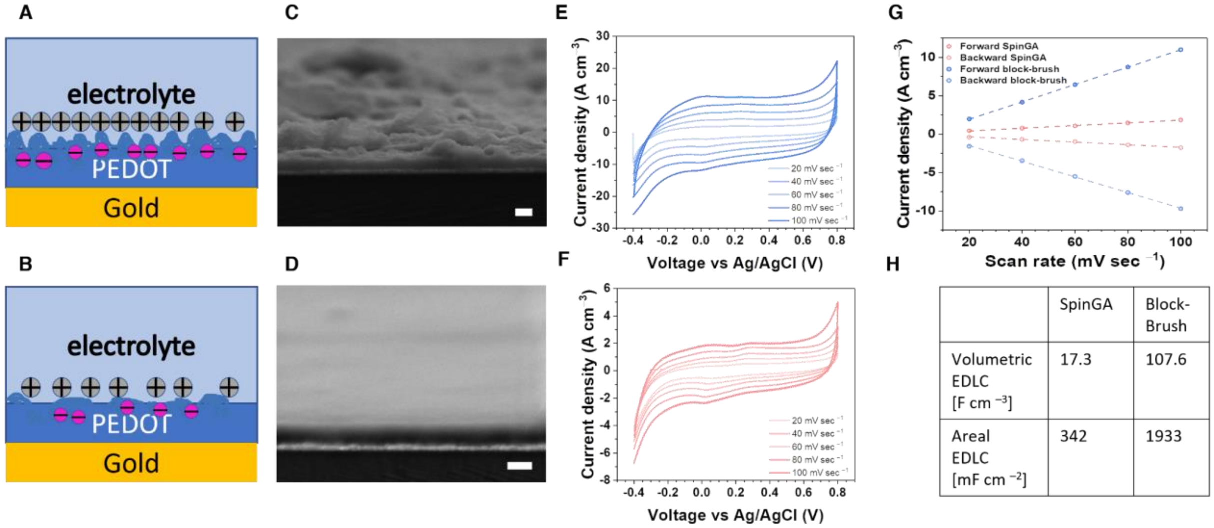

	SpinGA	Block-Brush
Volumetric EDLC [F cm⁻³]	17.3	107.6
Areal EDLC [mF cm⁻²]	342	1933

Figure S13. Block-brush film exhibits a higher volumetric electrical double layer (EDLC) in comparison with SC formulation. (**a, b**) Schematic illustration of the rougher surface of the block-brush film (**a**) and SpinGA film (**b**). (**c**) Representative SEM image of the block-brush film, that demonstrates its rough surface in comparison with SpinGA (**d**). The scale bars are 200 µm. (**e**) Cyclic voltammetry scans at low and different scan rates, showing greater charge storage capacity for the block-brush film and in comparison with the SpinGA film (**f**). (**g**) Current density comparison shows a linear increase in current density for increasing scan rate. The average of the slopes of the forward and background scan reveal the electric double layer capacitance (EDLC) of the block-brush and the SpinGA that is summarized in (**h**). Panel C is reproduced from Figure 1E to clearly show the different surfaces corresponding to block-brush (C) and SpinGA (D) films.

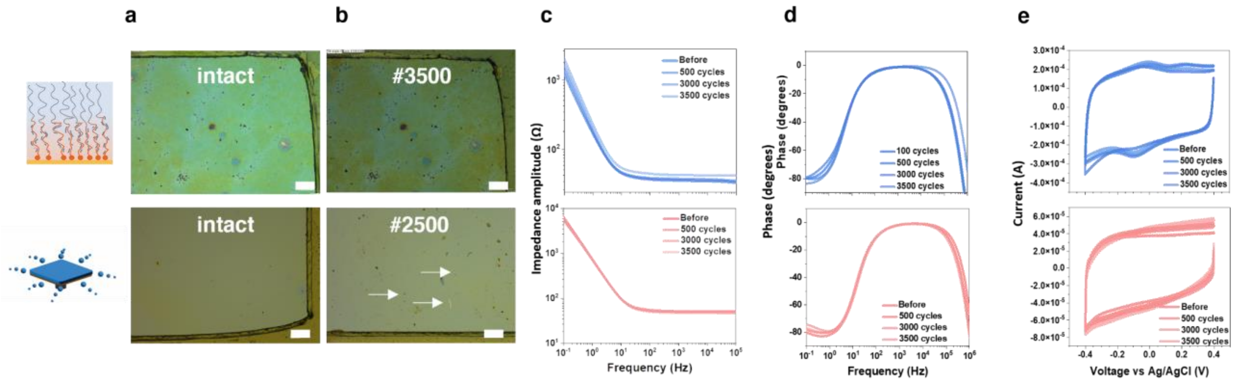

Figure S14. Block-brush film shows longer durability versus spin-coated PEDOT:PSS under CV cycling stress. (**a**) Optical microscope images taken during stability tests under cycling stress show the block-brush (upper panel) and SpinGA (lower panel) before (**a**) and (**b**) after CV cycles. No damage was detectable up to 3500 CV cycles for the block-brush film versus the damages that can be observed for the SpinGA film after 2500 CV cycles (lower panel, white arrows). (**c** to **e**) Corresponding Impedance magnitude (**c**) phase (**d**) spectra and (**e**) CV cycles. Scale bars are 250 µm. CV and EIS measurement continued up to 3500 cycles for both PEDOT-based films. Scale bars are 250 µm.

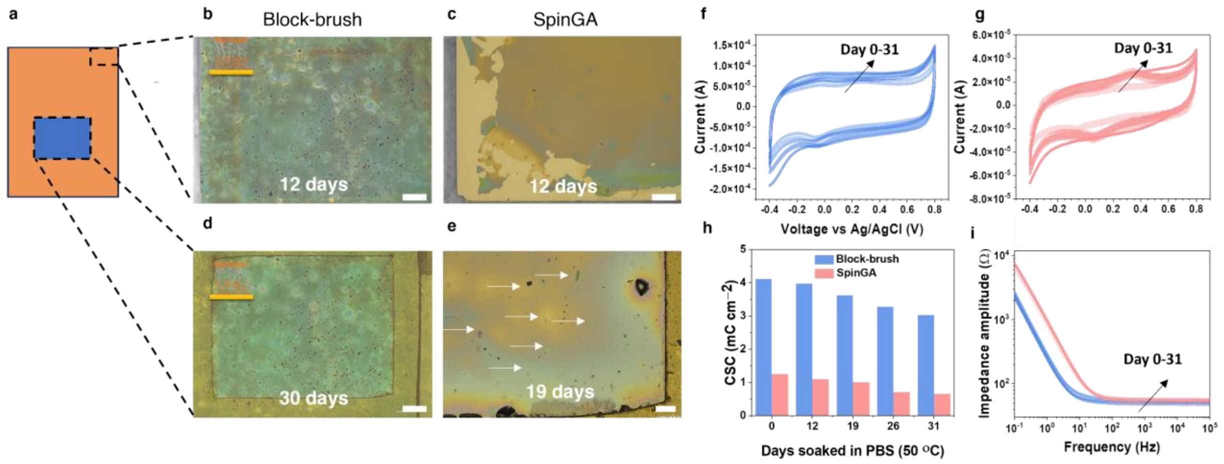

Figure S15. Block-brush films show increased stability on Au surfaces during accelerated aging test, in PBS, 50°C. (**a**) Schematic illustration of the Au surface covered with Kapton tape, exposing film area of 6 mm × 8 mm during an accelerated aging test (PBS, 50°C). (**b**) Optical microscope images of the edges of the coated surfaces that were covered by Kapton tape. PEDOT block-brush films are fully covering the area under the Kapton tape and the exposed rectangular (**b**), while the spin-coated film, SpinGA, was delaminated under the Kapton tape 12 days post incubation in PBS (50°C) (**c**). (**d**). The block-brush film is intact showing no damage under an optical microscope 30 days post incubation. The scale bars for B to D, are 1 mm. (**e**) The exposed area of the spin-coated PEDOT:PSS film had multiple delamination (white arrows) spots 19 days post-incubation. The scale bar is 250 µm. Accordingly, the CV cycling of the brushes (**f**, blue) showed a lower change in the CSC compared with the spin-coated films (**g**, pink) and (**h**). (**i**) The impedance stability of the block-brush and the SpinGA films soaked in PBS solution at 50°C for 26 days. The geometric surface area of the surfaces is 0.48 cm^2.

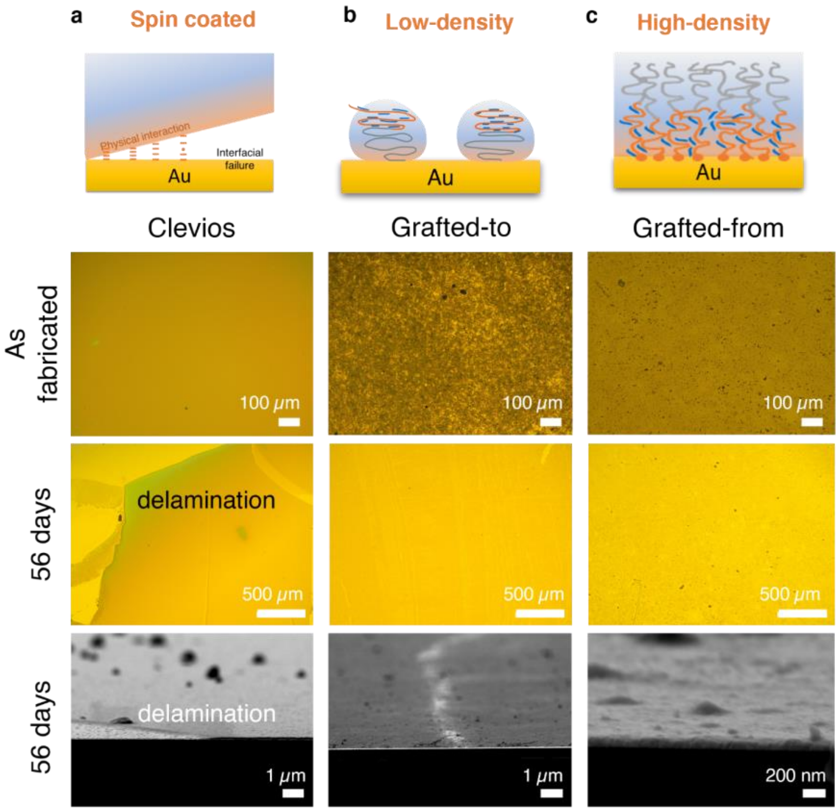

Figure S16. High-density block-brush PEDOT films show increased stability against accelerated aging test. Oxidative reactive accelerated aging test was done in 20mM H_2O_2 in PBS at 50°C for 56 days. (**a**) Spin-coated samples were delaminated from the surface. (**b**) Low-density brushes, grafted to the gold surface developed cracks in the film. (**c**) High-density block-brush films show an intact film with no observed damage through an optical microscope (two upper panels) or SEM (lower panel).

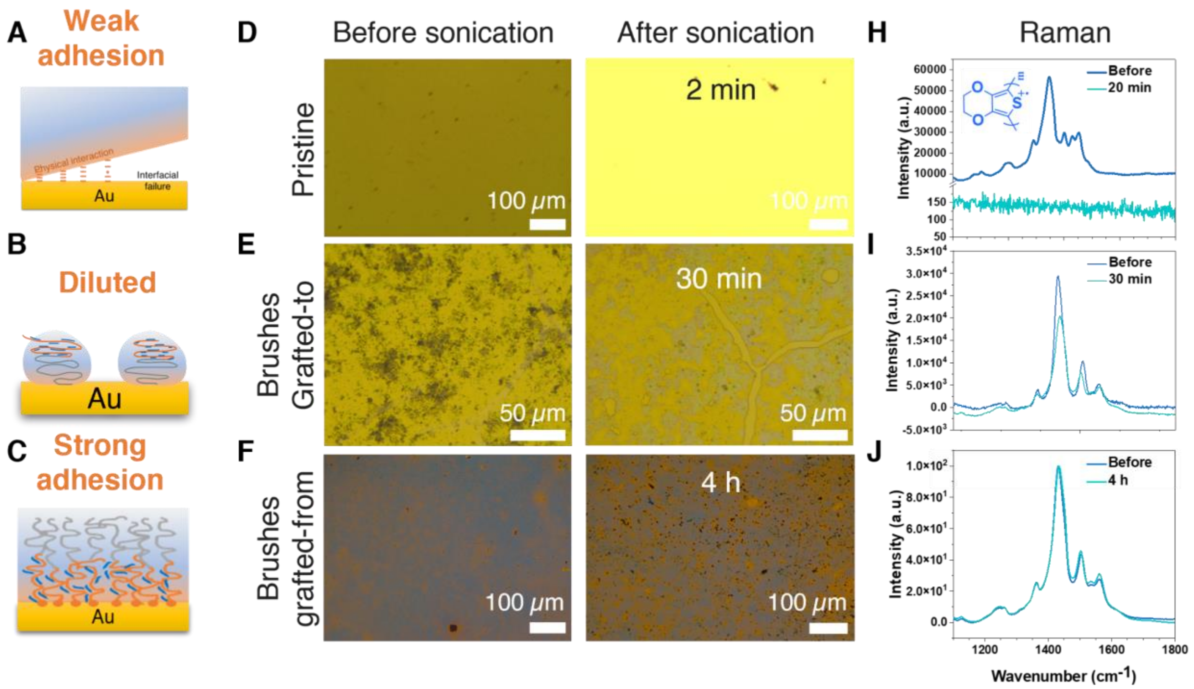

Figure S17. Mechanical stability of the bulk films. (**a**) Schematic illustration of the weak adhesion of spin-coated pristine PEDOT:PSS. (**b**) Schematic illustration of low-density block-brush, grafted to the gold surface. (**c**) Schematic illustration of strong adhesion of block-brush films. (**d** to **f**) Corresponding optical microscope images of the films before (left) and after (right) ultrasonication tests. The block-brush that was grafted from the surface showed the longest stability against sonication. The grafted-to films showed cracks 30 min post ultrasonication. (**h** to **j**) Corresponding Raman spectra before (blue) and after (cyan) ultrasonication, show complete PEDOT removal for the pristine sample after 2 min ultrasonication. Panels D, H, F, and J are reproduced from Figures 3B and 3C to show the differences between the block-brush grafted from the surface, the brushes grafted to the surface, and the Pristine PEDOT:PSS.

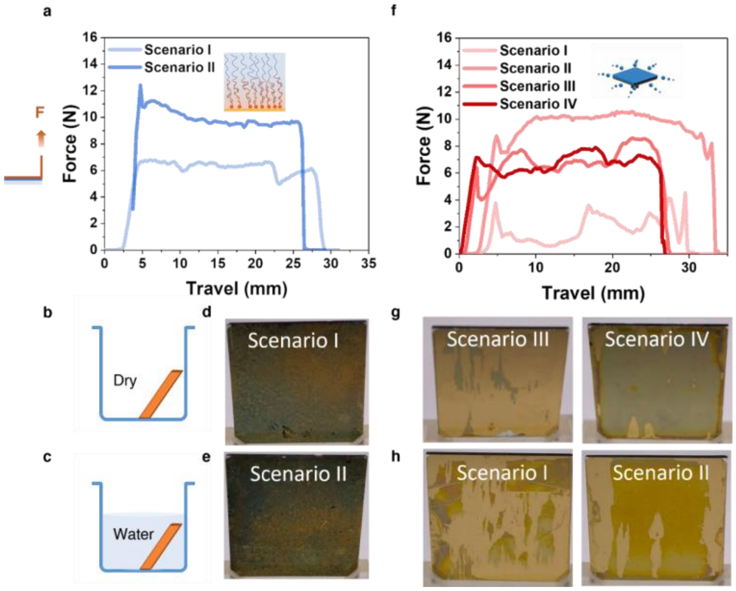

Figure S18. Mechanical stability of the bulk films. (**a**) 90° peel test samples were measured by peeling PEDOT-based films off using adhesive polyimide tape. (**b-d**) The block-brush films were not delaminated following the peel test for two evaluated scenarios. (**b** and **d**) when tested on dry film and when tested on dry film films that were previously soaked in water for 24 h (**c** and **e**). (**F**) The SpinGA, showed 4 different scenarios, with partial to substantial delamination for dry films (**b** and **g**) and for dry films that were previously soaked in water for 24 h (**c** and **h**). (**d** and **e**) Scenario I represents dry films that were not soaked in water before the peel test. Scenario II represents films that were soaked in water for 24 h and dried before the peel test. (**g** and **h**) Scenarios I and II represent films that were soaked in water for 24 h and dried before the peel test. Scenarios III and IV represent dry films that were not soaked in water before the peel test.

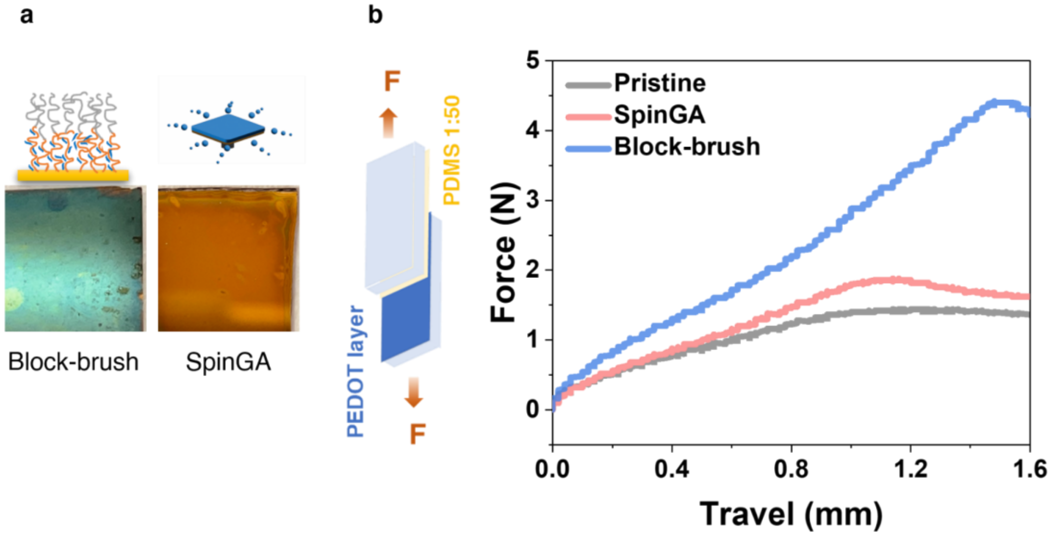

Figure S19. Mechanical stability of the bulk films. (**a**) Photographic images and corresponding illustrations of the block-brush films, and SpinGA films before mechanical tests. (**b**) Schematic illustration of a lap-joint shear test and the force applied perpendicular to the films versus the travel.

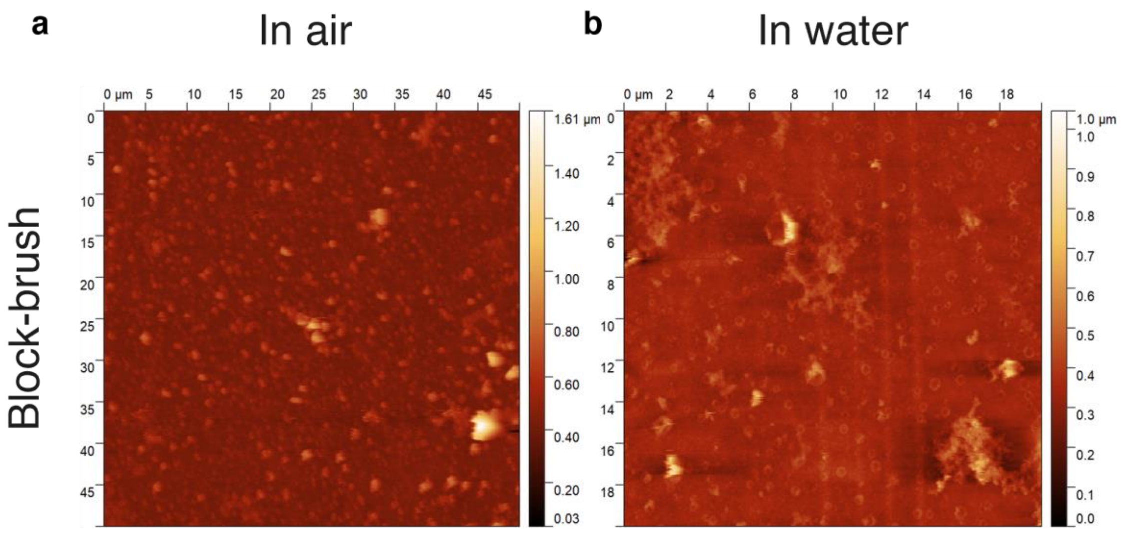

Figure S20. Dry (**a**) *versus* wet (**b**) Block-brush topology (height) obtained by AFM scans, showing full coverage of the surface.

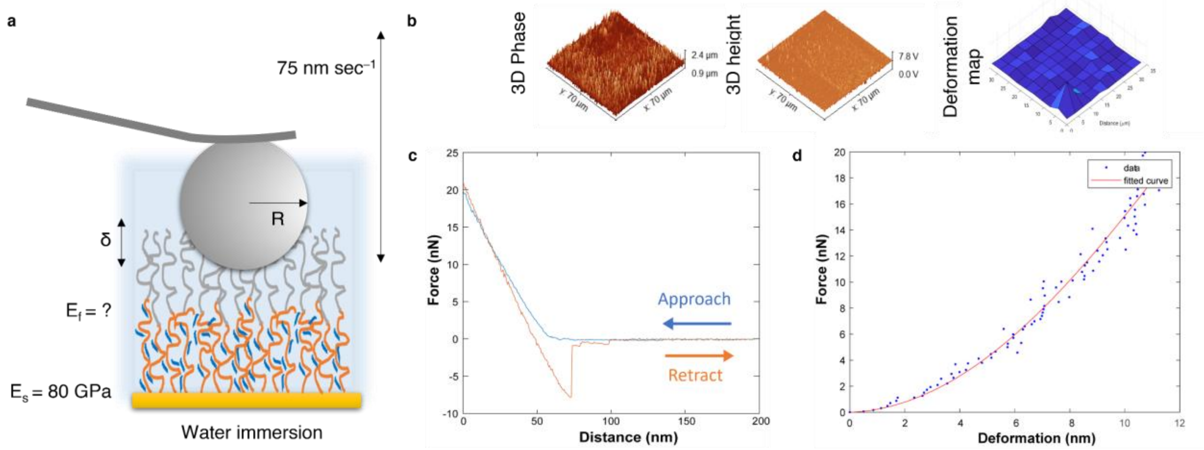

Figure S21. Calculating the elastic modulus of the block-brush film in water. (**a**) Schematic illustration of AFM tip-polymer brush interactions by nanoindentation. The interactions were analyzed using the Dimitriadis model of elastic deformation (Equations 5).[75] (**b**) 3D phase, height topography, and deformation map of the indentation area of block-brush PEDOT film. (**c**) A representative pull-off curve for the brushes, including arrows of the different interactions between the cantilever and the surface. (**d**) A representative force-deformation curve on the brushes. The force-deformation curves were fitted using the Dimitriadis model, by which the deformation map was generated and the Young's modulus in water was calculated.

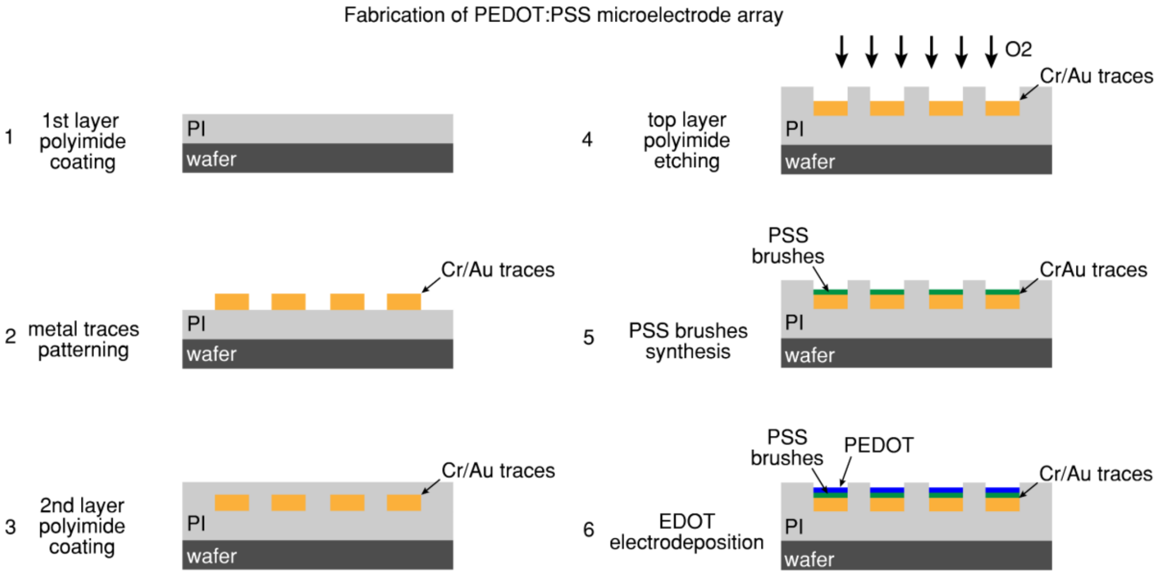

Figure S22. Fabrication process of microelectrode array coated with block-brush. (1) The first layer of PI was spin coated onto wafer surface. (2) Metal traces consisting of 10 nm Cr and 150 nm of Au were subsequently deposited by e-beam evaporation. (3) The second layer of PI was spin-coated onto the surface of the sample for encapsulation. (4) The via holes were etched using O2 plasma etching. (5) PSS-*b*-PPEGMEMA brushes were grafted from the Au surface as described in **Figure S3**. (6) PEDOT was electrodeposited on the polymer brush film as described in section VI.

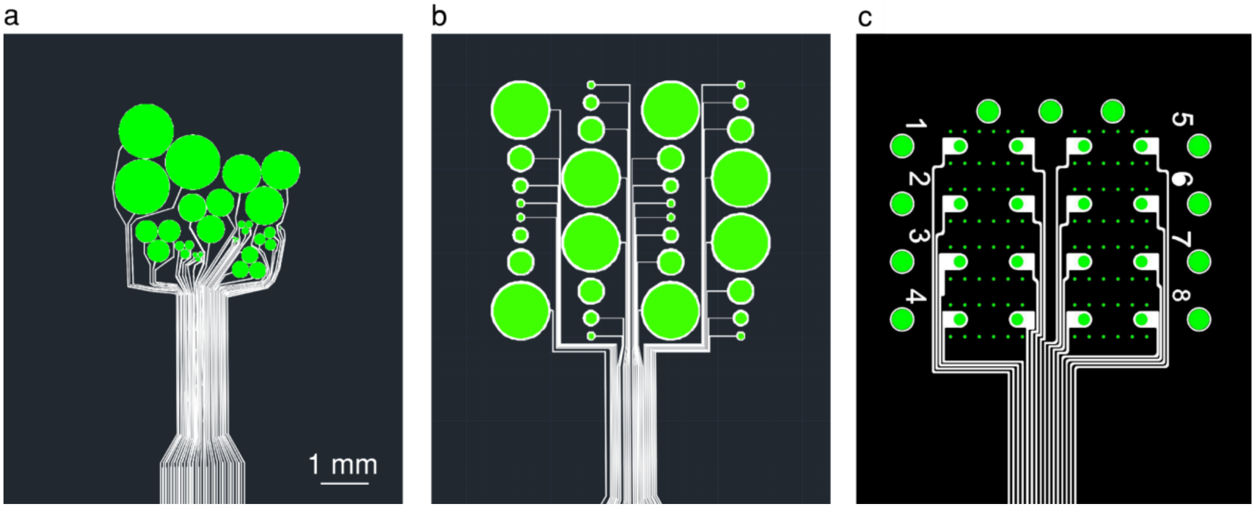

Figure S23. Multidiameter microelectrode array design. (**a**) Version 1 of the benchtop multidiameter array. (**b**) Version 2 of the benchtop multidiameter array with 8 x 1000 µm, 8 x400 µm, 8 x 200 µm, and 8 x 100 µm diameter contacts. (**c**) The rat microelectrode array with 16 x 200 µm diameter contacts.

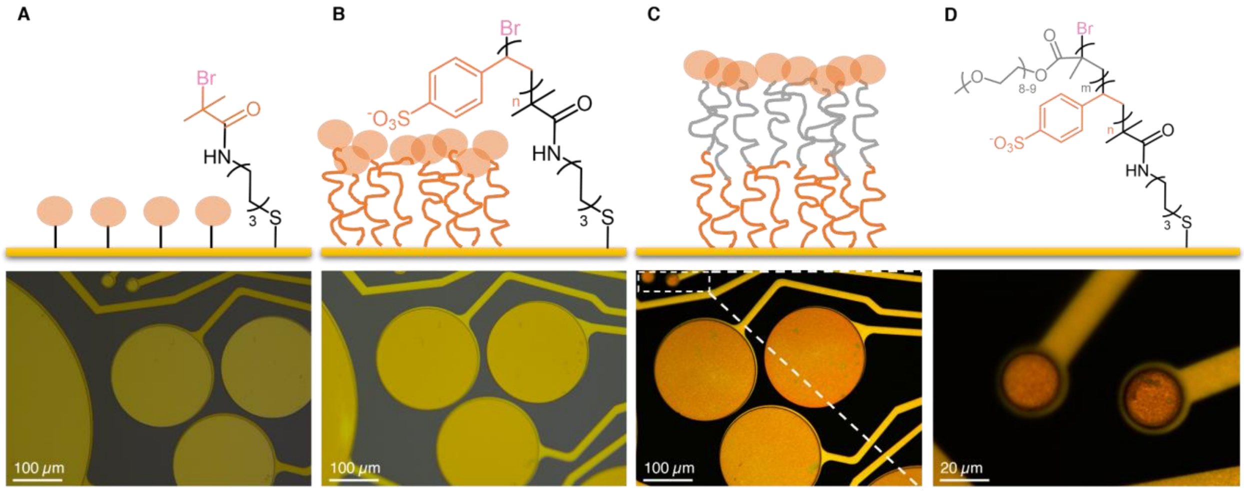

Figure S24. Polymer brushes grafted from the Au surfaces on the multi-diameter electrode. Optical microscope image of the Au surface after the activation with ATRP initiator (**a**), modification with PSS brushes (**b**), and further modification with the second block copolymer PPEGMEMA (**c**). **d**. The inset shows the uniformity on the 20 µm diameter electrode.

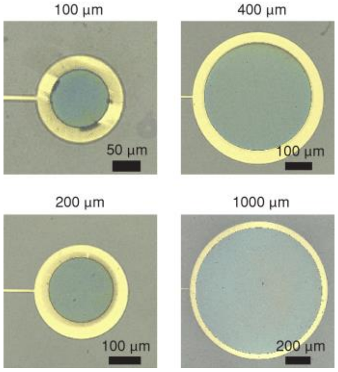

Figure S25. Example of block-brush coated contacts of the multidiameter array.

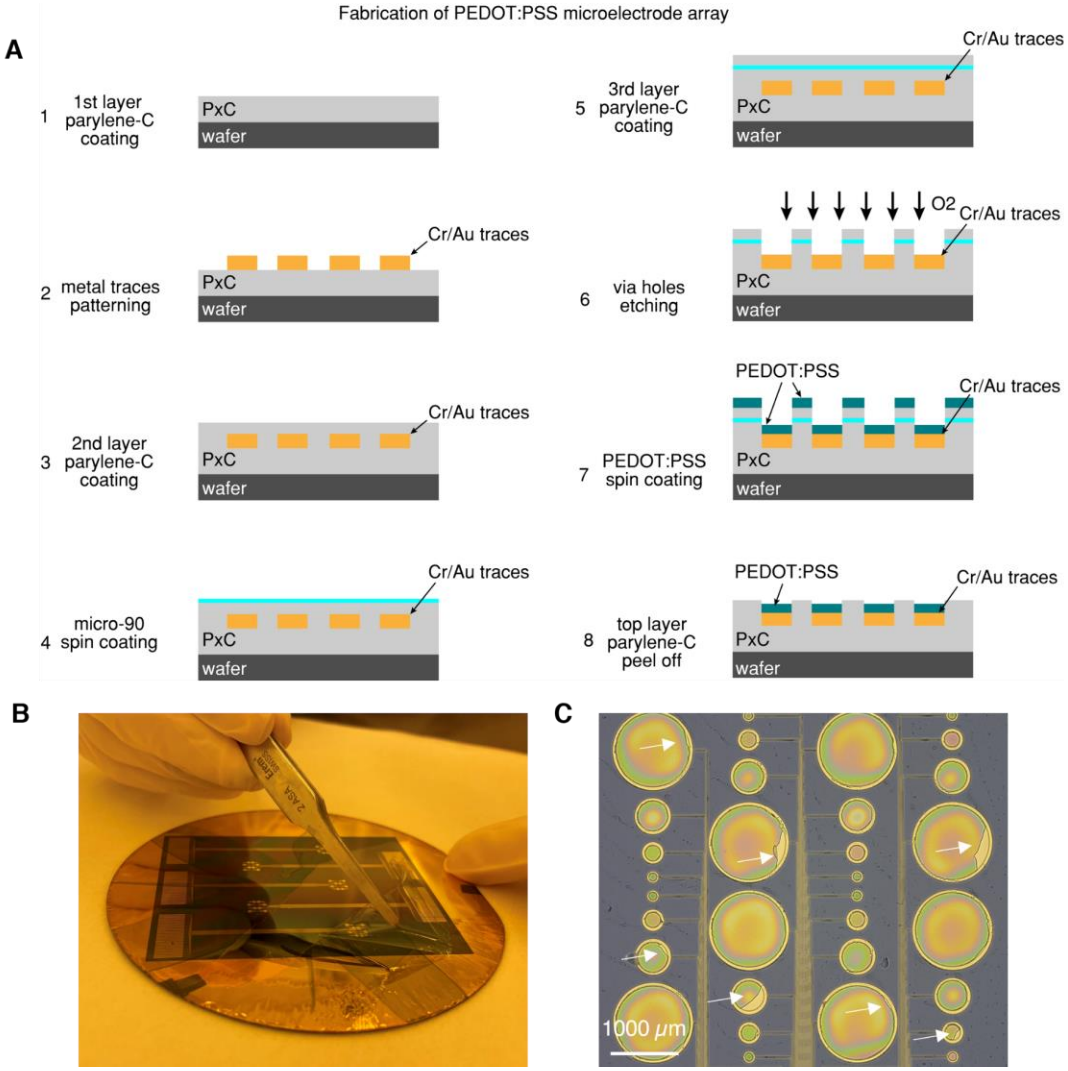

Figure S26. Fabrication process of spin-coated PEDOT:PSS control microelectrode array. (**a**) The process is similar to the preparation of block-brush coating, but requires additional steps needed for the spin-coated sample preparation. These include the addition of a third sacrificial layer of parylene C and then peeling it off to create the PEDOT:PSS pattern on the gold circular contacts only. (**b**) Picture of the additional required to peel-off step to remove the 3rd layer of the parylene C coating and retain the spin coating PEDOT:PSS layer only on the gold contacts. (**c**) Optical microscope image demonstrating some damaged contacts (white arrows) resulting from the peel-off process off the 3rd layer of the parylene C coating.

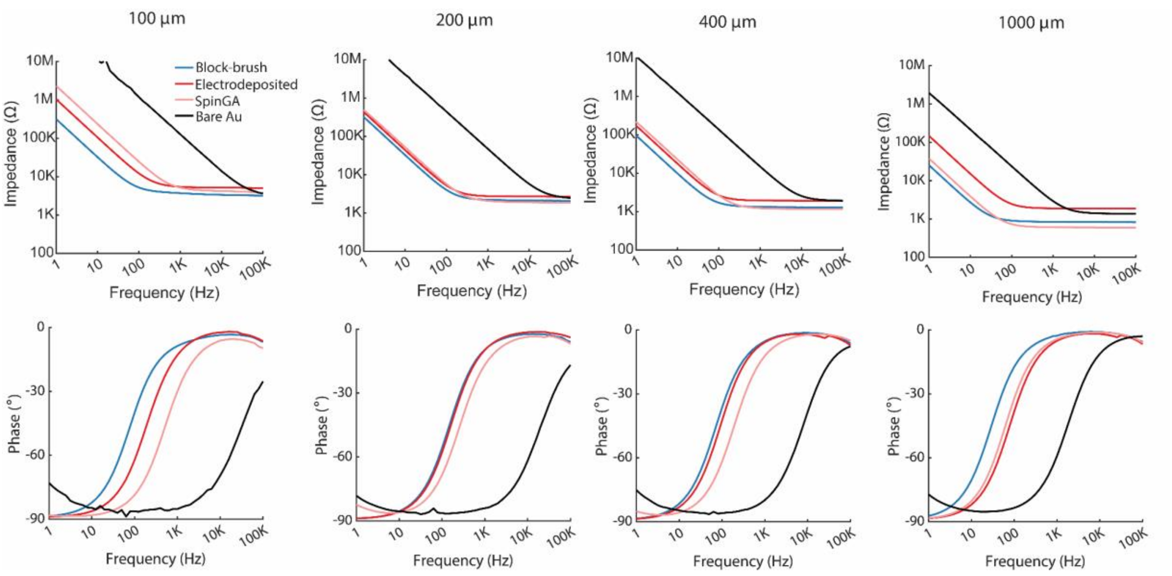

Figure S27. Impedance spectra of different contacts diameters after electrodeposition. The block-brush films (blue) show the lowest impedance under 100Hz for all diameters, in comparison with the spin coated formulation and the electrodeposited control.

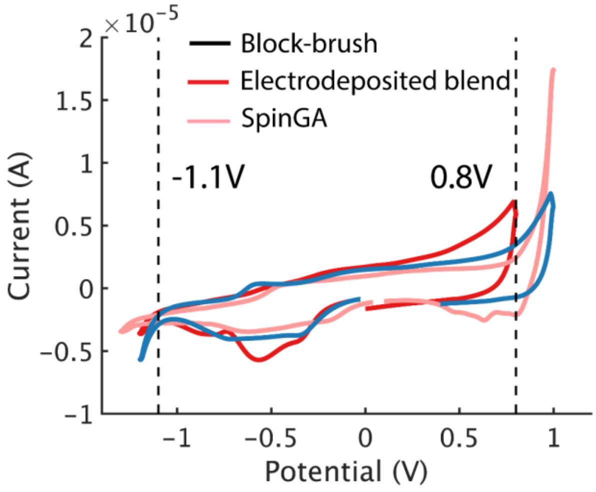

Figure S28. Water window of the 1000 µm diameter array for the three PEDOT formulations. The water window of the block-brush between –1.1V and 0.8V is marked by the dashed lines.

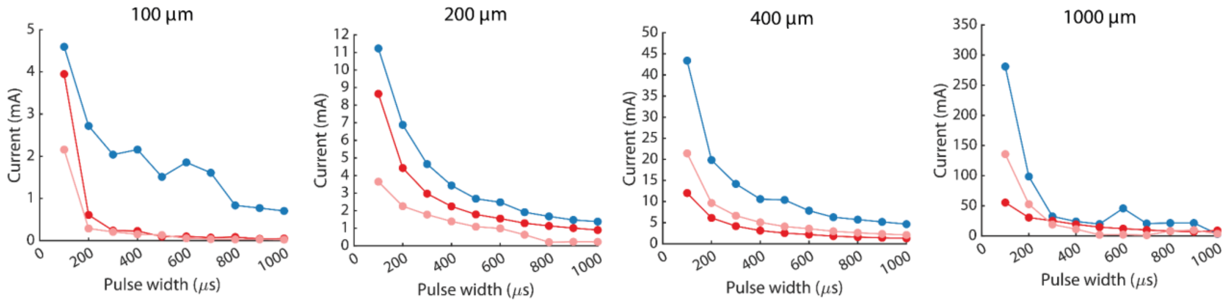

Figure S29. CIC for the three PEDOT:PSS materials different contacts diameters.

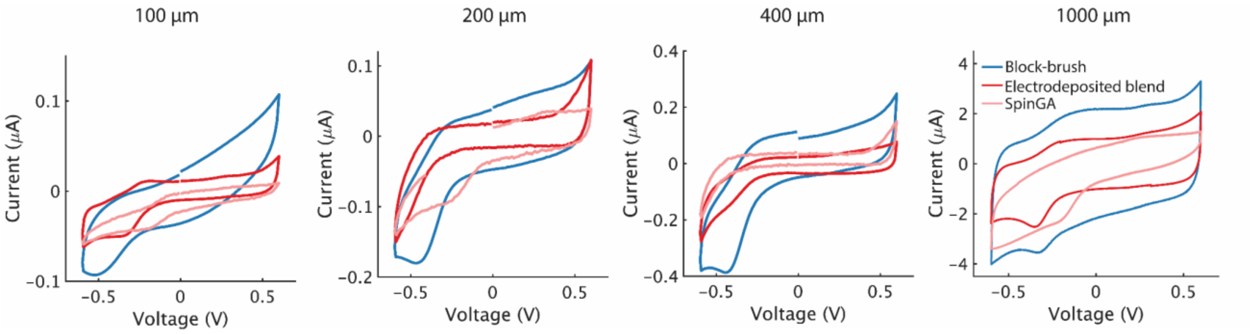

Figure S30. CV of different contacts diameters after electrodeposition. The block-brush films (blue) demonstrate the largest AUC, hence the highest CSC in comparison with the spin coated formulation and the electrodeposited control.

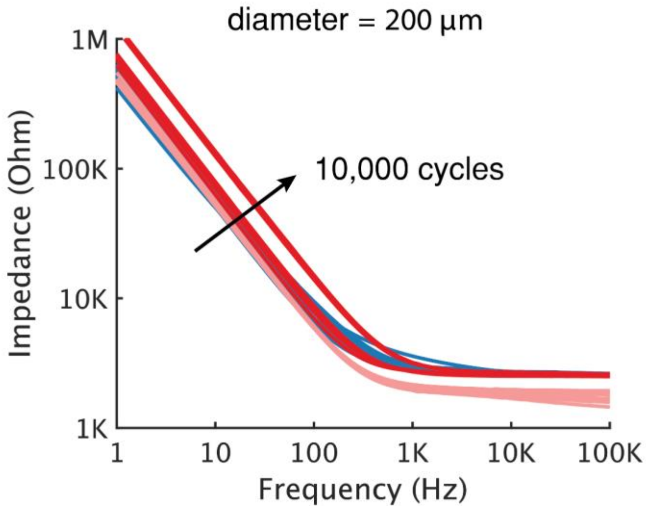

Figure S31. Stability of spin coated, electrodeposited and block-brush film under electrochemical cycling. EIS measurements show the stability of the films over 10,000 CV cycles.

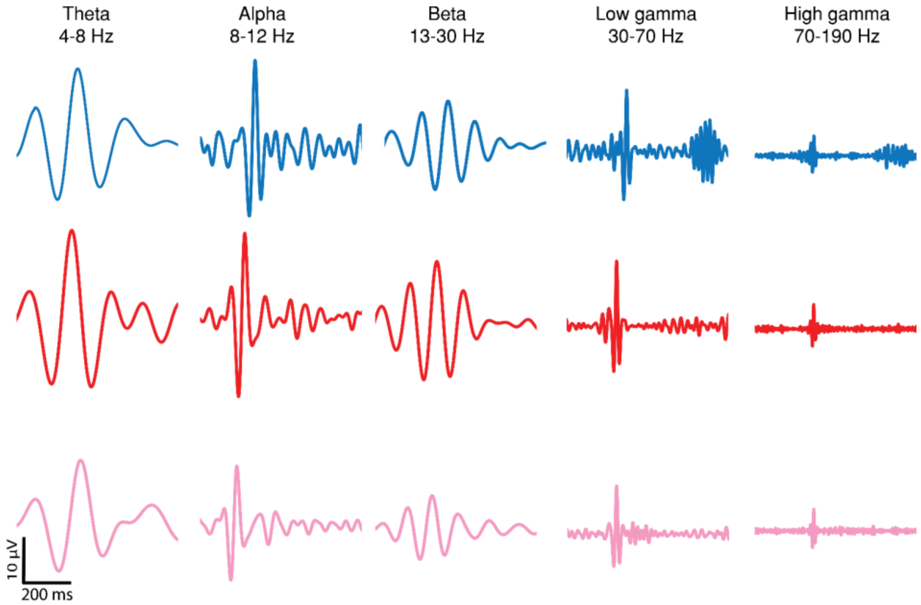

Figure S32. Spectral analysis of the mean trial-averaged response across channels to whisker air puff stimulation. Across frequency bands, responses from the three materials showed similar spectral profiles. mean trial-averaged response across channels filtered by frequency band for the block-brush (top, blue), electrodeposited control (middle, red), and spin coated control (bottom, pink) samples. This indicates that the modified PEDOT:PSS material can capture all frequency components of neural activity.

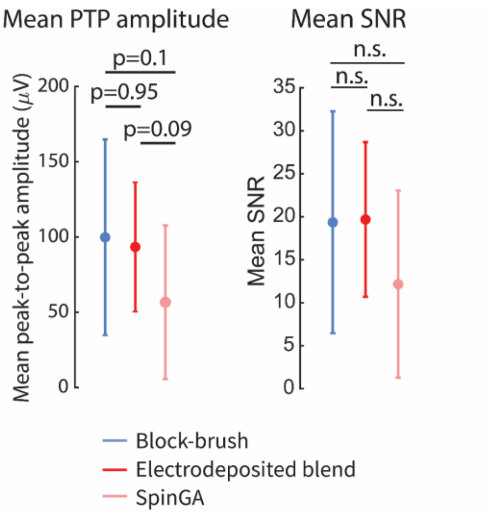

Figure S33. Mean peak-to-peak (PTP) and SNR of the different PEDOT based films. The block-brush film on the gold contact of the multi-array electrode showed the highest PTP. P denoted the statistical significance obtained. The statistical significance of the peak-to-peak was evaluated by the Mann-Whitney U test, using Matlab (ranksum function, n = 6 for the electrodeposited blend and the block-brush, n = 7 for the SpinGA).
